# Systematic review of machine learning applications using nonoptical motion tracking in surgery

**DOI:** 10.1038/s41746-024-01412-1

**Published:** 2025-01-14

**Authors:** Teona Z. Carciumaru, Cadey M. Tang, Mohsen Farsi, Wichor M. Bramer, Jenny Dankelman, Chirag Raman, Clemens M. F. Dirven, Maryam Gholinejad, Dalibor Vasilic

**Affiliations:** 1 Department of Plastic and Reconstructive Surgery, Erasmus MC University Medical Center, Rotterdam, the Netherlands; 2 Department of Neurosurgery, Erasmus MC University Medical Center, Rotterdam, the Netherlands; 3 Medical Library, Erasmus MC University Medical Center, Rotterdam, the Netherlands; 4 Department of Biomechanical Engineering, Delft University of Technology, Delft, the Netherlands; 5 Department of Pattern Recognition and Bioinformatics, Delft University of Technology, Delft, the Netherlands

**Keywords:** Outcomes research, Health services, Surgery

## Abstract

This systematic review explores machine learning (ML) applications in surgical motion analysis using non-optical motion tracking systems (NOMTS), alone or with optical methods. It investigates objectives, experimental designs, model effectiveness, and future research directions. From 3632 records, 84 studies were included, with Artificial Neural Networks (38%) and Support Vector Machines (11%) being the most common ML models. Skill assessment was the primary objective (38%). NOMTS used included internal device kinematics (56%), electromagnetic (17%), inertial (15%), mechanical (11%), and electromyography (1%) sensors. Surgical settings were robotic (60%), laparoscopic (18%), open (16%), and others (6%). Procedures focused on bench-top tasks (67%), clinical models (17%), clinical simulations (9%), and non-clinical simulations (7%). Over 90% accuracy was achieved in 36% of studies. Literature shows NOMTS and ML can enhance surgical precision, assessment, and training. Future research should advance ML in surgical environments, ensure model interpretability and reproducibility, and use larger datasets for accurate evaluation.

## Introduction

Machine learning (ML) models have gained consistent attention within the medical field for their potential to revolutionise healthcare practices. ML algorithms are adept at modelling high dimensional data distributions, improving process efficiency, and reducing burden on healthcare professionals through data-driven insights^1,2^. They can be trained to identify data patterns and optimise predictive precision^3–5^, making them valuable tools in medical decision-making across various specialties, such as radiology^5,6^ and oncology^7^. This successful integration of ML into healthcare workflow demonstrates how technology to complement and enhance the capabilities of medical experts.

An emerging domain for ML application is surgical motion tracking, which offers potential advancements in surgical practice. Capturing and analysing the motion characteristics of surgeons’ hands and surgical instruments during procedures provides valuable data for several purposes. Surgical skill training and evaluation are labour-intensive and time-consuming for both trainers and trainees. Their automation could offer much-needed efficiency^8,9^, support professional development, and ensure high-quality care. Additionally, motion data could aid the development of assistive surgical tools to improve surgeon precision and patient outcomes. Research has also explored using surgical motion data to predict patient post-surgical outcomes^10^, offering the potential for real-time adjustments during surgery to reduce post-operative complications.

However, much of the existing surgical motions tracking research relies on visual sensors, such as cameras. While these systems are valuable for their convenience and integration into laparoscopic and robotic surgical devices, they have inherent limitations, such as poor quality and susceptibility to occlusion^11^. Non-optical motion tracking systems (NOMTS) offer promising solutions by providing robust and versatile data capture capabilities without the constraints of optical systems.

This systematic review aims to provide an overview of ML applications in surgical manoeuvre analysis using NOMTS. Objectives include identifying ML algorithms and models used, comparing their effectiveness, identifying NOMTS applications in surgical settings, and highlighting research trends, gaps, challenges, and future research directions.

## Results

### Search results

**Fig. 1:**
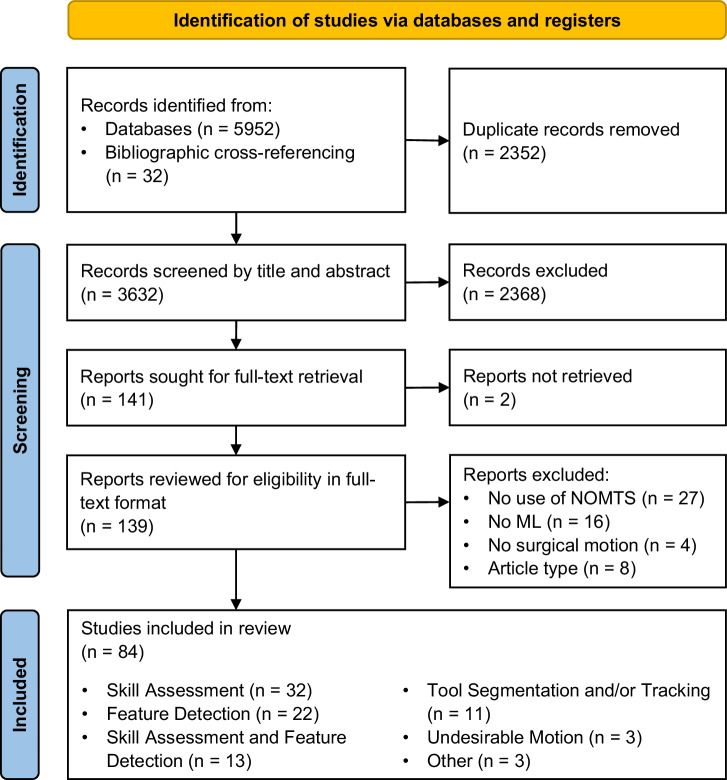
PRISMA flow diagram of study inclusion process. The figure shows the number of records identified, retrieved, assessed, and included at different stages within the systematic review process. From 3632 unique records, 84 studies were included. The 84 studies are further divided into their respective categories of ML aims.

A total of 3632 unique records were identified through the literature search after duplicate removal. An additional 32 records were identified by bibliographic cross-referencing. After undergoing screening based on title and abstract, as well as full-text retrieval, a total of 139 studies were assessed in full text. The inclusion process led to 84 reports meeting the criteria for inclusion (Fig. 1). Table 1 provides full overview of the included studies, categorised by their machine learning aim. Six primary machine learning aims were identified: (1) skill assessment (SA); (2) feature detection (FD); (3) a combination of skill assessment and feature detection; (4) tool segmentation and/or tracking (TT); (5) undesirable motion filtration (UMF); (6) other. These are further detailed in the Results sections *ML tasks*.

**Table 1:** Overview of included studies, categorised in order of machine learning aim

Index	Author	Year	Sensor	Video	Field	Task	Subjects	Trials	Machine Learning Model	Performance Metric (%)	Cross-Validation
**Skill Assessment**											
1	Ahmidi, N^72^.	2015	EM	CO	Open	CM	14	86	(Stroke-based) SVM	MA: 74.24-90.91	LOTO, LOUO
									Descriptive Curve Coding + SVM	MA: 81.03-91.66	
									HMM + SVM	MA: 23.06-70.93	
2	Albasri, S^12^.	2021	DK (J)	CO	Robotic	BB	10	150	Procrustes DTW + kNN	MA: 88.9-100	LOSO
			I	No	Open	CS	4	120	Procrustes DTW + kNN	MA: 80-100	LOSO, LOTO
3	Allen, B^70^.	2010	EM	No	Lap.	BB	30	696	SVM	MA: 90-93.7	Hold out
4	Baghdadi, A^50^.	2020	DK + M	No	Robotic	BB	30	1440	LASSO + RF	MA: 63	k-fold
									LASSO + kNN	MA: 63	
									LASSO + LR	MA: 70	
									LASSO + RF + kNN + LR	MA: 78	
5	Bissonnette, V^46^.	2019	DK	No	Open	CS	41	41	SVM	MA: 97.6	LOO, k-fold
									kNN	MA: 92.7	
									LDA	MA: 87.8	
									Naive Bayes	MA: 86.9	
									Decision tree	MA: 70.7	
6	Brown, J.D^85^.	2017	I + M	CO	Robotic	BB	38	110	SVM + Elastic Net Regression + Regression Trees + kNN	MA: 63.3-73.3	LOO
									RF	MA: 51.7-75	
7	Brown, K.C^32^.	2020	DK	CO	Robotic	CM	-	100-131	LR	MA: 76.32-98.27	k-fold
8	Chen, A.B^39^.	2021	DK	CO	Robotic	CM	17	68	RF	MA: 71.6-76.9	-
									AdaBoost	MA: 69.9-80.1	
									Gradient Boosting	MA: 67.2-78.4	
9	Fard, M.J^53^.	2018	DK (J)	CO	Robotic	BB	8	80	kNN	MA: 71.9-89.7	LOSO, LOUO
									LR	MA: 70.2-89.9	
									SVM	MA: 75.4-79.8	
10	Horeman, T^92^.	2012	M	No	Lap.	BB	31	93	PCA + LDA	MA: 78-84	LOO
11	Hung, A.J^38^.	2018	DK	No	Robotic	CM	9	78	RF	MA: 87.2	Stratified k-fold
									SVM	MA: 83.3	
									LR	MA: 82.1	
12	Hung, A.J^10^.	2019	DK	No	Robotic	CM	8	100	MLP (DeepSurv)	-	k-fold
13	Hung, A.J^68^.	2022	DK	CO	Robotic	BB	22	226	NoiseRank + LSTM	-	-
14	Jiang, J^73^.	2017	EM	CO	Robotic	BB	10	10	DTW	-	-
15	Jog, A^67^.	2011	DK	No	Robotic	BB	17	41	Decision tree + SVM	MA: 67.5-87.5	k-fold
16	Kelly, J.D^40^.	2020	DK	CO	Lap.	BB	117	454	Bi-LSTM	MA: 73.33-96.88	Hold out
17	Khan, A^86^.	2020	I	CO	Open	BB	15	50	SVM	-	LOTO, LOUO, k-fold
18	Laverde, R^88^.	2018	I	No	Lap.	BB	7	207	ANN	-	k-fold
19	Li, K^51^.	2020	DK (J)	No	Robotic	BB	-	96	kMC + DNN	ME: 9.18-9.47	-
20	Lin, Z^89^.	2011	I	No	Lap.	BB	16	48	PCA + LDA	MA: 93.75	LOO
21	Lin, Z^87^.	2013	I	No	Lap.	BB	16	96	PCA + LDA	MA: 94	LOO
22	Lyman, W.B^52^.	2021	DK	No	Robotic	CS	2	25	Kernel Regularised Linear Squares Multivariate prediction + Multivariate Linear Regression	MA: 89.3	-
23	Megali, G^48^.	2006	DK	No	Lap.	BB	6	24	HMM	-	Hold out
24	Oquendo, Y.A^71^.	2018	EM + M	CO	Lap.	BB	32	63	Regularised Least Squares + Regression Trees	MA: 38-88	LOUO
25	Sbernini, L^90^.	2018	I + M	No	Open	BB	18	360	LDA	ME:5.86-8.06	LOO
									SVM	ME: 0.89-2.05	
									MLP	ME: 0.57-0.61	
26	Sewell, C^69^.	2008	DK	CO	Open	CS	15	30	HMM	MA: 87.5	LOO
									Naive Bayes	-	
									LR	MA: 50-100	
27	Soangra, R^13^.	2022	I + EMG	No	Open + Lap. + Robotic	BB	26	234	RF	MA: 40-60	Hold out
									Naive Bayes	MA: 28-47	
									SVM	MA: 35-57	
28	Uemura, M^41^.	2018	EM	No	Lap.	BB	67	67	Chaotic NN	MA: 79	Hold out
29	Wang, Z.H^43^.	2018	DK (J)	CO	Robotic	BB	8	40	CNN	MA: 84.9-95.4	LOSO, Hold out
30	Watson, R.A^91^.	2014	I	No	Other	CS	24	48	SVM	MA: 83	-
31	Xu, J^93^.	2023	M	No	Open	BB	13	20	LSTM	MA: 76.67-78.86	LOUO
									Bi-LSTM	MA: 80.51-84.92	
									GRU	MA: 75.46-77.57	
									Convolutional LSTM DNN	MA: 93.65-96.19	
									Transformer network	MA: 86.68-90.67	
									TCN	MA: 88.95-97.45	
32	Zhang, D^20^.	2020	DK	Yes	Robotic	BB	8	66	CNN	MA: 84.72-97.92	LOSO
			DK (J)	CO	Robotic	BB	8	103	CNN	MA: 80.80-99.17	LOSO
**Feature Detection**											
33	Ahmidi, N^21^.	2017	DK (J)	CO	Robotic	BB	8	101	LDA + GMM-HMM	MA: 64.12-92.56	LOSO, LOUO
									K-Singular Value Decomposition + Sparse-HMM	MA: 62.48-83.54	
									Markov semi-Markov CRF	MA: 44.68-81.99	
									Skip Chain CRF	MA: 74.77-85.18	
									Linear Dynamical System	MA: 47.96-84.61	
			DK (J)	Yes	Robotic	BB	8	101	Markov semi-Markov CRF	MA: 65.87-85.1	LOSO, LOUO
									Skip Chain CRF	MA: 81.60-85.04	
34	van Amsterdam, B^63^.	2019	DK (J)	CO	Robotic	BB	8	40	GMM	MA: 59-85	Experimental Validation
35	van Amsterdam, B^45^.	2020	DK (J)	CO	Robotic	BB	8	39	Bi-LSTM	MA: 85.1-89.2	LOUO
36	van Amsterdam, B^22^.	2022	DK (J)	Yes	Robotic	BB	8	39	CNN + Concatenation TCN	MA: 82.3	LOUO
									CNN + Ensemble TCN	MA: 82.6	
									CNN + Multimodal Attention TCN	MA: 83.4	
			DK	Yes	Robotic	CM	8	45	CNN + Concatenation TCN	MA: 79.3	Hold out
									CNN + Ensemble TCN	MA: 78.1	
									CNN + Multimodal Attention TCN	MA: 80.9	
37	Despinoy, F^61^.	2016	DK	CO	Robotic	BB	3	12	kNN	MA: 78.4-97.4	LOO
									SVM	MA: 77.5-96.2	
38	DiPietro, R^14^.	2019	DK	CO	Robotic	BB	15	39	RNN	ME: 17.9	LOUO
									LSTM	ME: 15.3	
									GRU	ME: 15.2	
									MIST RNN	ME: 15.3	
			DK (J)	CO	Robotic	BB	8	39	RNN	ME: 11.6	LOUO
									LSTM	ME: 8.7	
									GRU	ME: 8.6	
									MIST RNN	ME: 9.7	
39	Fard, M.J^64^.	2016	DK (J)	CO	Robotic	BB	8	-	PCA + DTW + Soft-Boundary Unsupervised Gesture Segmentation	MA: 64-73.8	Experimental Validation
40	Gao, Y^23^.	2016	DK (J)	CO	Robotic	BB	8	39	DTW + Autoencoder	MA: 68-84	-
			DK	CO	Robotic	BB	15	55	DTW + Autoencoder	MA: 59-74	-
41	Goldbraikh, A^81^.	2022	EM	CO	Open	BB	24	96	MS-TCN + +	MA: 82.4-94.69	k-fold
									LSTM	MA: 79.94-94.18	
									GRU	MA: 82.21-95.04	
42	Goldbraikh, A^24^.	2024	EM	CO	Open	BB	25	11	Bi-LSTM MS-TCRN	MA: 83-84.2	k-fold
									Bi-GRU MS-TCRN	MA: 83.1-84.3	
			EM	CO	Open	CM	52	255	Bi-LSTM MS-TCRN	MA: 77.8-80.5	LOUO
									Bi-GRU MS-TCRN	MA: 77.4-79.2	
			DK (J)	CO	Robotic	BB	8	39	Bi-LSTM MS-TCRN	MA: 84.2-84.8	LOUO
									Bi-GRU MS-TCRN	MA: 85.0-86.4	
43	Itzkovich, D^25^.	2019	DK (J)	CO	Robotic	BB	8	39	LSTM	MA: 67-72	LOUO
			DK	CO	Robotic	BB	2	14	LSTM	MA: 55-71	LOUO
44	Itzkovich, D^26^.	2022	DK (J)	CO	Robotic	BB	8	75	LSTM	MA: 46-64	Hold out
			DK	CO	Robotic	BB	2	15	LSTM	MA: 8-52	Hold out
			DK	CO	Robotic	CM	6	-	LSTM	MA: 13-68	Hold out
45	Lea, C^65^.	2016	DK (J)	CO	Robotic	BB	8	39	Latent Convolutional Skip Chain CRF	MA: 81.69-83.45	LOUO
46	Lin, H.C^54^.	2006	DK	No	Robotic	BB	2	27	LDA + Bayes Classifier	MA: 92.21-95.26	k-fold
47	Long, Y^27^.	2021	DK (J)	Yes	Robotic	BB	8	75	CNN + TCN-LSTM + Graph NN	MA: 87.9-88.1	LOUO
			DK	Yes	Robotic	BB	-	36	CNN + TCN-LSTM + Graph NN	MA: 87.3-91.0	k-fold
48	Loukas, C^75^.	2013	EM	CO	Lap.	CS	21	21	Gaussian mixture MAR	-	-
49	Meißner, C^84^.	2014	I + EM	CO	Other	CS	2	24	HMM	MA: 81-99	LOO
50	Murali, A^66^.	2016	DK (J)	Yes	Robotic	BB	8	67	PCA + CNN + GMM + Transition state clustering	-	-
51	Peng, W^62^.	2019	DK	CO	Robotic	BB	12	360	DTW + Continuous HMM	MA: 94.73-97.48	Experimental Validation
52	Qin, Y^28^.	2020	DK (J)	Yes	Robotic	BB	8	39	CNN-TCN + LSTM-TCN	MA: 86.3	LOUO
			DK	Yes	Robotic	CM	5	10	CNN-TCN + LSTM-TCN	MA: 82.7	LOUO
53	Zheng, Y^74^.	2022	EM	CO	Lap.	BB	29	29	LSTM	MA: 68.18-75.86	LOUO
54	Zia, A^37^.	2019	DK	Yes	Robotic	CM	-	100	CNN-LSTM + LSTM	-	Hold out
**Skill Assessment and Feature Detection**											
55	Anh, N.X^55^.	2020	DK (J)	No	Robotic	BB	8	40	CNN + SVM	MA: 92.75-96.84	LOSO
									LSTM + SVM	MA: 89.75-95.09	
									CNN-LSTM + SVM	MA: 90.98-96.39	
									Autoencoder + SVM	MA: 80.63-83.46	
56	Baghdadi, A^36^.	2023	M	No	Open	CM	13	50	CNN + DNN-LSTM	MA FD: 82-95	k-fold
									KNN + XGBOOST + DNN-LSTM	MA SA: 71	
57	Ershad, M^76^.	2019	EM	CO	Robotic	BB	14	84	PCA + SVM	MA: 71.03-98.5	k-fold
58	Forestier, G^15^.	2018	DK (J)	CO	Robotic	BB	8	101	SAX-VSM	MA FD: 75.29-93.69	LOSO, LOUO
										MA SA: 61.11-96.3	
			DK	No	Robotic	BB	3	30	SAX-VSM	MA FD: 100	LOO
										MA SA: 83.33	
			DK	CO	Robotic	CS	6	27	SAX-VSM	MA SA: 85.19	LOO
59	King, R.C^16^.	2009	I + M	No	Lap.	BB	5	25	HMM	MA FD: 56-100	-
			I + M	No	Lap.	CM	7	28	PCA + HMM + GMM Clustering	-	-
60	Loukas, C^77^.	2011	EM	CO	Lap.	BB	22	44	MAR + PCA + SVM	MA: 86-96	-
									HMM	MA: 65-87	
61	Loukas, C^78^.	2013	EM	CO	Lap.	CS	22	22	MAR	-	-
62	Nguyen, X.A^17^.	2019	I	CO	Open	BB	15	75	SVM	MA: 71.3-81.7	LOSO
									CNN-LSTM + SVM	MA: 88.1-95.4	
									CNN-LSTM + SENet + SVM	MA: 90.3-96.7	
									CNN-LSTM + SENet + Restart + SVM	MA: 92.1-98.2	
			DK (J)	No	Robotic	BB	8	101	CNN-LSTM + SVM	MA: 91.5-97.3	LOSO
									CNN-LSTM + SENet + SVM	MA: 94.7-98.3	
									CNN-LSTM + SENet + Restart + SVM	MA: 94.8-98.4	
63	Reiley, C.E^60^.	2010	DK	CO	Robotic	BB	11	20	DTW + GMM/GMR + HMM	-	-
64	Rosen, J^33^.	2001	M	CO	Lap.	CM	10	10	kMC + HMM	MA: 87.5	-
65	Topalli, D^49^.	2019	DK	No	Other	BB	28	1260	kNN + AdaBoost M1	MA: 85.71	k-fold
									kNN + Jrip	MA: 64.28-78.57	
									kNN + kNN	MA: 57.14-75	
									kNN + Locally Weighted Learning	MA: 67.86-82.14	
									kNN + LR	MA: 75-82.14	
									kNN + SVM	MA: 64.28-82.14	
66	Wang, Z^44^.	2018b	DK (J)	CO	Robotic	BB	8	120	GRU-CNN	MA FD: 100	LOSO
										MA SA: 96	
67	Zia, A^18^.	2018	I	CO	Open	BB	41	103	ApEn + Cross ApEn + Nearest Neighbour	MA: 78.7-86.8	k-fold, LOO
			I	Yes	Open	BB	41	103	kMC + ApEn + Cross ApEn + Nearest Neighbour	MA: 93.2-94	k-fold, LOO
**Tool Tracking**											
68	Korte, C^47^.	2021	DK	No	Open	CS	5	60	LSTM-RNN	-	Experimental validation
69	Lee, E.J^19^.	2019	EM	Yes	Lap.	BB	-	1500	Random walk + Deep CNN	-	Hold out
			EM	Yes	Lap.	CM	-	100	Random walk + Deep CNN	-	-
70	Liu, J^34^.	2023	DK	Yes	Robotic	CM	-	950	CNN	-	LOO
71	Pachtrachai, K^30^.	2021	DK	Yes	Robotic	BB	-	8502	CNN + LSTM	-	Experimental validation
			DK	Yes	Robotic	CM	-	15002	CNN + LSTM	-	Experimental validation
72	Qin, Y^29^.	2020	DK (J)	Yes	Robotic	BB	8	39	CNN-LSTM + LSTM Encoder + LSTM Decoder	ME: 4.72-10.14	LOUO
			DK	Yes	Robotic	CM	5	40	CNN-LSTM + LSTM Encoder + LSTM Decoder	ME: 1.1-2.43	LOUO
73	Rocha, C.D^31^.	2019	DK	Yes	Robotic	BB	-	910	GMM + CNN	MA: 99	Experimental validation
			DK	Yes	Robotic	BB	-	2737	GMM + CNN	MA: 98.2	Experimental validation
			DK	Yes	Robotic	CM	-	481	GMM + CNN	MA: 97	Experimental validation
74	Shu, X^56^.	2021	DK	No	Robotic	NCS	-	1524	MLP	ME: <1.5	Hold out
									LSTM	ME: <1.5	
75	Sun, Z^83^.	2018	EM	No	Other	NCS	-	150	ANN	-	Experimental validation
76	Wang, Z^82^.	2022	EM	No	Lap.	BB	4	80	LSTM	ME: 11.43-15.11	Hold out
77	Xu, W^79^.	2017	EM	No	Robotic	NCS	-	20000	GMR	MA: 87.39-95	Hold out
									kNN	MA: 90.5-95.9	
									Extreme machine learning	MA: 98.2	
78	Zhao, H^59^.	2018	DK (J)	Yes	Robotic	BB	8	67	PCA + DTW + Transition State Clustering Dense Convolutional Encoder-Decoder Network	MA: 60.1-70.6	LOO
**Undesirable Motion Filtration**											
79	Sang, H^57^.	2016	I + DK	No	Other	NCS	-		Zero Phase Adaptive Fuzzy Kalman Filter	-	Experimental validation
80	Tatinati, S^95^.	2015	I	Yes	Other	NCS	3	6	Moving Window Least Squares - SVM	MA: 71	Experimental validation
81	Tatinati, S^94^.	2017	I	Yes	Other	NCS	3	9	Moving Window Least Squares - SVM	MA: 74	Experimental validation
									Multidimensional Robust Extreme Learning Machine	MA: 78	
									Online sequential Multidimensional Robust Extreme Learning Machine	MA: 81	
**Other**											
82	Sabique, P.V^35^.	2023	M + DK	Yes	Robotic	BB	-	-	PCA + Generalised Discriminant Analysis + RNN-LSTM	-	Experimental validation
									PCA + Generalised Discriminant Analysis + CNN-LSTM	-	
									PCA + GDA + Encoder network	-	
83	Song, W^80^.	2006	M + EM	Yes	Open	BB	-	120	Fuzzy NN	-	-
84	Su, H^58^.	2019	M + DK	No	Robotic	NCS	-	73776	ANN	-	Experimental validation

An overview of the methodologies and technologies employed across different studies.

**Sensor:** *DK* device kinematics, *(J)* JHU-ISI Gesture and Skill Assessment Working Set dataset, *I* inertial*, EM* electromagnetic*, M* mechanical*, EMG* electromyography. **Video:** *CO* context only. **Field:** *Lap*. Laparoscopic. **Task:** *BB* basic bench-top, *CS* clinical simulation, *NCS* non-clinical simulation, *CM* clinical model. **Machine Learning Model:** *SVM* support vector machine, *HMM* hidden Markov model, *DTW* dynamic time warping, *kNN* k-nearest neighbours, *LASSO* least absolute shrinkage and selection operator, *RF* random forest, *LR* logarithmic regression, *LDA* linear discriminant analysis, *PCA* principal component analysis, *MLP* multilayer perceptron, *LSTM* long short-term memory, *Bi-* bidirectional, *ANN* artificial neural network, *kMC* k-means clustering, *DNN* deep neural network, *NN* neural network, *CNN* convolutional neural network, *GRU* gated recurrent unit, *TCN* temporal convolutional network, *GMM* Gaussian mixture model, *CRF* conditional random field, *MIST* mixed history, *RNN* recurrent neural network, *MS* multi-stage, *TCRN t*emporal convolutional recurrent network, *SAX-VSM* symbolic aggregate approximation vector space model, *MAR* multivariate autoregressive*, SENet* squeeze-and-excitation network, *GMR* Gaussian mixture regression, *ApEn* approximate entropy. **Performance Metric:** *MA* mean accuracy, *ME* mean error. **Cross Validation:** *LOTO* leave one trial out, *LOUO* leave one user out, *LOSO* leave one super-trial out, *LOO* leave one out.

### Data collection and sources

**Fig. 2:**
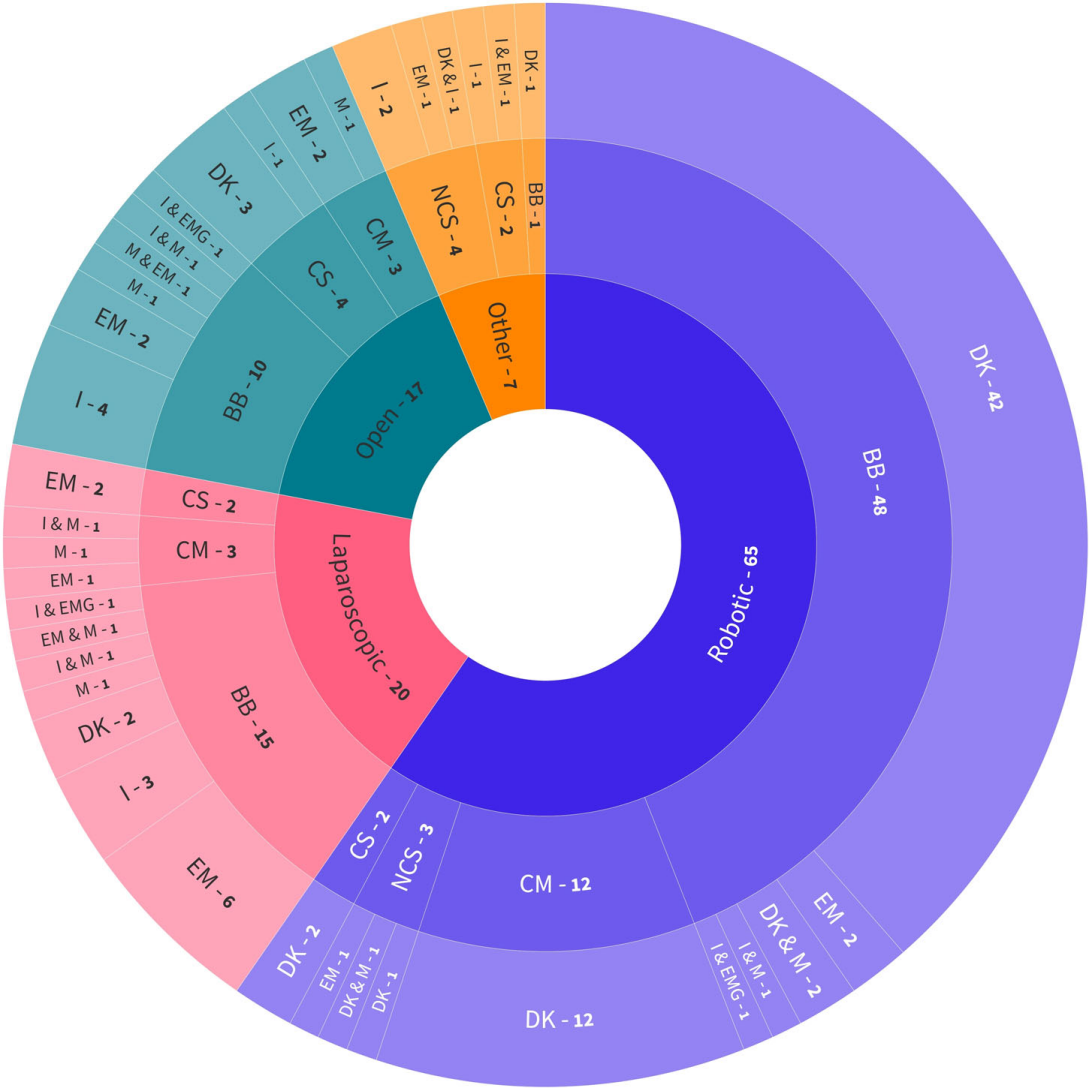
Experiment configurations of the included studies. Central layer represents surgical field. Middle layer represents task type. External layer represents sensor types and combinations: *DK* device kinematic, *EM* electromagnetic, *I* inertial, *M* mechanical, *EMG* electromyography.

The included studies featured one or more experiments, each designed with different set-ups, sensors, and procedures. Twenty studies included more than one experiment^12–31^. The procedures were categorised by surgical field and task. Robotic procedures were the most common, appearing in 65 experiment types, followed by laparoscopic in 20, and open in 17. Basic bench-top (BB) tasks, such as peg transfer or suturing, composed 72 experiments. Clinical simulations (CS), which mimic real-life surgery, were conducted in 10 experiments. Clinical models (CM) were used in 18 experiments, including animal models^19,24,26,28,29,31–35^, cadaver models^16,29,34^, and real-life surgeries like septoplasty^12^, tumour removal^36^, or prostatectomy^10,22,30,37–39^. Non-clinical simulations (NCS), which simulate surgical movement without a defined surgical task, were present in eight experiments (Fig. 2).

Among the experiments with human participants, 40 utilised datasets with at least 10 participants, while only 14 included at least 25 participants (Table 1). The largest datasets included 117 participants^40^, followed by 67 participants^41^ and 52 participants^24^.

One frequently used public dataset was the JHU-ISI Gesture and Skill Assessment Working Set (JIGSAWS)^42^, which appeared in 26 use cases (Table 1). It includes synchronised robotic video and tool motion from eight surgeons performing BB tasks (needle passing, knot tying, suturing) within a robotic surgical context. Multiple studies leveraged this dataset to compare their algorithms with others on the same dataset^15,17,21,22,24–28,43–45^, as well as for transfer learning applications^20,26^.

Another dataset, used by two studies, is the Johns Hopkins Minimally Invasive Surgical Training and Innovation Center Science of Learning Institute (MISTIC-SL) dataset^14,23^. It consists of synchronised robotic video and tool motion during BB tasks. The Robotic Intra-Operative Ultrasound (RIOUS) and RIOUS+ datasets are used by Qin et al. containing robotic video and tool motion of drop-in ultrasound scanning in dry-lab, cadaveric, and in-vivo settings^28,29^. The Basic Laparoscopic Urologic Skills (BLUS) also features synchronised video and tool motion of BB laparoscopic tasks^40^. The Bowel Repair Simulation (BRS) dataset consists of 255 porcine open enterotomy repair procedures captured with electromagnetic sensors and two camera views^24^. However, these datasets are not publicly available.

### Non-optical motion tracking systems (NOMTS)

The included studies utilised five categories of NOMTS across various experiments, often featuring multiple experiment types within a single study. In total, 107 experiment designs were found across the 84 studies.

1. Device kinematic (DK) data recordings: in 67 experiments to capture the internal position logging of virtual reality^46,47^, laparoscopic^40,48^, endoscopic^49^, or robotic^10,12,14,15,17,20–32,34,35,37–39,43–45,50–69^ surgical devices.
2. Electromagnetic (EM) systems: in 20 experiments, mostly using active EM systems^19,24,41,70–82^, except for a passive magnetic system^83^ and radio frequency identification (RFID)^84^.
3. Inertial (I) sensors: in 18 experiments, including accelerometers^12,13,16,18,84–89^ and inertial measurement units^17,57,90,91^.
4. Mechanical (M) sensors: in 13 experiments, including force^33,35,36,50,58,80,85,92,93^ and flex sensors^16,71,90^.
5. Surface electromyography (EMG): in one study^13^.

Twelve experiments combined multiple NOMTS types^13,16,35,50,57,58,71,80,84,85,90^, with mechanical^16,35,50,58,71,80,85,90^ and inertial^13,16,57,84,85,90^ sensors being the most frequently combined types. All combinations of experimental designs may be found in Fig. 2.

### Optical sensor data as an NOMTS supportive tool

Of the 81 experiment designs that did not use optical sensors as input for ML analysis, 47 used video recordings to provide context for NOMTS data processing. The video recording served several purposes, including providing time-stamps, enabling third-party expertise evaluation, contextualising non-visual data, and facilitating manual annotation of manoeuvres and gestures. Twenty-six experiments incorporated additional optical sensors for ML analysis, including red-green-blue (RGB) endoscopic cameras^34,37,39,40,44,45,68^, RBG cameras aimed the subject^18,20,24,35,69,80^, and infrared (IR) cameras^94,95^. Among these, 19 experiments required manual annotation^34,35,37,59,66,80^. However, five experiments aimed to train their algorithms to automatically segment image frames, using their annotations as ground truth verification^31,59,66^. Two studies trained their ML models exclusively on optical data before testing on NOMTS data^94,95^ (Table 2).

**Table 2:** Optical data collection types and purpose in included studies

Index	Author	Year	Optical Type	Purpose
1	Ahmidi, N^72^.	2015	Kinect (RGB and IR)	Annotate tool usage times
2	Ahmidi, N^21^.	2017	Robotic endoscope video	Annotate gesture type
			Robotic endoscope video	Model training and validation
3	Albasri, S^12^.	2020	Robotic endoscope video	Grade skill level
4	van Amsterdam, B^63^.	2019	Robotic endoscope video	Annotate gesture type
5	van Amsterdam, B^45^.	2020	Robotic endoscope video	Annotate gesture type
6	van Amsterdam, B^22^.	2022	Robotic endoscope video	Annotate gesture type; Model training and validation
		2022	Robotic endoscope video	Annotate gesture type; Model training and validation
7	Brown, J.D^85^.	2017	Robotic endoscope video	Grade skill level
8	Brown, K.C^32^.	2020	Robotic endoscope video	Annotate start/stop times of tasks
9	Chen. A.B^39^.	2021	Robotic endoscope video	Annotate start/stop times of tasks
10	Despinoy, F^61^.	2016	Robotic endoscope video	Annotate gesture type
11	DiPietro, R^14^.	2019	Robotic endoscopic video	Annotate manoeuvre type
			Robotic endoscopic video	Annotate gesture type
12	Ershad, M^76^.	2019	Videos of subject, video of task	Crowdsourced stylistic labelling
13	Fard, M.J^64^.	2016	Robotic endoscope video	Annotate gesture type
14	Fard, M.J^53^.	2018	Robotic endoscope video	Grade skill level
15	Forestier, G^15^.	2018	Robotic endoscope video	Annotate gesture type
			Robotic endoscope video	Annotate gesture type
16	Gao, Y^23^.	2016	Robotic endoscope video	Annotate gesture type
			Robotic endoscope video	Annotate gesture type
17	Goldbraikh, A^81^.	2022	Videos of subject, video of task	Annotate tool usage and gesture type
18	Goldbraikh, A^24^.	2024	Video of subject, video of task	Annotate gesture and manoeuvre type
			Video of subject, video of task	Annotate gesture and manoeuvre type
			Robotic endoscope video	Annotate gesture and manoeuvre type
19	Hung, A.J^68^.	2022	Robotic endoscope video	Annotate manoeuvre type; Grade skill level
20	Itzkovich, D^25^.	2019	Robotic endoscope video	Annotate gesture type
			Robotic endoscope video	Annotate gesture type
21	Itzkovich, D^26^.	2022	Robotic endoscope video	Annotate gesture type
			Robotic endoscope video	Annotate gesture type
			Robotic endoscope video	Annotate gesture type
22	Jiang, J^73^.	2017	Robotic endoscope video	Annotate instrument trajectories; Annotate start/stop times of tasks
23	Kelly, J.D^40^.	2020	Laparoscopic video	Grade skill level (via expert and crowdsourcing)
24	Khan, A^86^.	2020	Video of subject	Annotate gesture type; Grade skill level
25	Lea, C^65^.	2016	Robotic endoscope video	Annotate gesture type
26	Lee, E.J^19^.	2019	Laparoscopic video	Model training and validation
			Laparoscopic video	Model training and validation
27	Liu, J^34^.	2023	Robotic endoscope video	Annotation of tools; Model training and validation
28	Long, Y^27^.	2021	Robotic endoscope video	Annotate gesture type; Model training and validation
			Robotic endoscope video	Annotate gesture type; Model training and validation
29	Loukas, C^75^.	2013	Video of task	Annotate manoeuvre type
30	Loukas, C^77^.	2011	Video of task	Assistance in interpretation of signals
31	Loukas, C^78^.	2013	Video of task	Assistance in interpretation of signals; Annotate gesture type
32	Meißner, C^84^.	2014	Video of instrument tray, video of task	Annotate active tool usage times; Annotate gesture type
33	Murali, A^66^.	2016	Robotic endoscope video	Annotate gesture type; Model training and validation
34	Nguyen, X.A^17^.	2019	Video of task	Grade skill level
35	Oquendo, Y.A^71^.	2018	Video of subject	Grade skill level
36	Pachtrachai, K^30^.	2021	Robotic endoscope video	Annotation of tools; Model training and validation
			Robotic endoscope video	Annotation of tools; Model training and validation
37	Peng, W^62^.	2019	Robotic virtual reality video	Annotate gesture type
38	Qin, Y^28^.	2020	Robotic endoscope video	Annotate gesture type; Model training and validation
			Robotic endoscope video	Annotate gesture type; Model training and validation
39	Qin, Y^29^.	2020	Robotic endoscope video	Annotate gesture type; Model training and validation
			Robotic endoscope video	Annotate gesture type; Model training and validation
40	Reiley, C.E^60^.	2010	Robotic endoscope video	Annotate manoeuvre type
41	Rocha, C.D^31^.	2019	Robotic endoscope video	Annotation of tools; Model training and validation
			Robotic endoscope video	Annotation of tools; Model training and validation
			Robotic endoscope video	Annotation of tools; Model training and validation
42	Rosen, J^33^.	2001	Video of task	Annotate gesture type
43	Sabique, P.V^35^.	2023	Video of task	Annotate tool motion; Model training and validation
44	Sewell, C^69^.	2008	Video of task	Grade skill level
45	Song, W^80^.	2006	Video of task	Model training and validation
46	Tatinati, S^94^.	2017	IR stylus	Model training
47	Tatinati, S^95^.	2015	IR stylus	Model training
48	Wang, Z.H^43^.	2018	Video of subject	Grade skill level
49	Wang, Z^44^.	2018	Robotic endoscope video	Annotate gesture type
50	Zhang, D^20^.	2020	Microscope video, video of task	Annotate tool motion; Model training and validation
			Robotic endoscope video	Annotate manoeuvre type
51	Zhao, H^59^.	2018	Robotic endoscope video	Annotation of tools; Model training and validation
52	Zheng, Y^74^.	2022	Video of task	Grade skill level; Error and peg transfer counting; Annotate frames as “stressed” or “normal”
53	Zia, A^18^.	2018	Video of task	Annotate manoeuvre type
			Video of task	Model training and validation
54	Zia, A^37^.	2019	Endoscopic video	Model training and validation

An overview of the optical data collection methods employed in the included studies, detailing their specific purposes within the experimental models.

### NOMTS sensor placement

Sensor placement varied across tasks within the studies, as detailed in Table 3, with studies exploring relevant sensor placement combinations for their tasks. One study highlighted the significance of shoulder joint metrics for laparoscopic skill assessment^87^, while another identified the most relevant sensors in a tracking glove for gesture and skill identification during tissue dissection tasks^16^. Additionally, another used an ML model to determine optimal EMG sensor placement for open, laparoscopic, and robotic tasks^13^.

**Table 3:** Included study sensor types, placement, and surgeon handedness inclusivity

Index	Author	Year	Sensor Types	Sensor Placement	Single /Double-handed	Left-handed (n)
1	Ahmidi, N^72^.	2015	EM	1 EM on tool, 1 EM on patient head	Single	–
2	Ahmidi, N^21^.	2017	DK, RGB cam.	Internal device recordings	Double	No
3	Albasri, S^12^.	2020	DK	Internal device recordings	Double	No
			Accelerometer	1 accelerometer per wrist	Double	Yes (1)
4	Allen, B^70^.	2010	EM	2 EM per laparoscopic arm	Double	–
5	van Amsterdam, B^63^.	2019	DK	Internal device recordings	Double	No
6	van Amsterdam, B^45^.	2020	DK	Internal device recordings	Double	No
7	van Amsterdam, B^22^.	2022	DK, RGB cam.	Internal device recordings	Double	No
			DK, RGB cam.	Internal device recordings	Double	–
8	Anh, N.X^55^.	2020	DK	Internal device recordings	Double	No
9	Baghdadi, A^50^.	2020	DK, Force	Internal device recordings, 1 force sensor between robotic end-effector and forceps	Single	–
10	Baghdadi, A^36^.	2023	Force	Force sensing bipolar forceps	Single	–
11	Bissonnette, V^46^.	2019	DK	Internal device recordings	Double	–
12	Brown, J.D^85^.	2017	Accelerometer, Force	1 accelerometer per robotic arm, 1 accelerometer on camera arm; 1 force sensor under working surface	Double	Yes (3)
13	Brown, K.C^32^.	2020	DK	Internal device recordings	Double	–
14	Chen, A.B^39^.	2021	DK	Internal device recordings	Double	–
15	Despinoy, F^61^.	2016	DK	Internal device recordings	Double	–
16	DiPietro, R^14^.	2019	DK	Internal device recordings	Double	No
					Double	No
17	Ershad, M^76^.	2019	EM	1 EM per shoulder, wrist, hand	Double	–
18	Fard, M.J^64^.	2016	DK	Internal device recordings	Double	No
19	Fard, M.J^53^.	2018	DK	Internal device recordings	Double	No
20	Forestier, G^15^.	2018	DK	Internal device recordings	Double	No
					Double	No
					Double	–
21	Gao, Y^23^.	2016	DK	Internal device recordings	Double	No
			DK	Internal device recordings	Double	No
22	Goldbraikh, A^81^.	2022	EM	1 EM per thumb, index, dorsal wrist	Double	No
23	Goldbraikh, A^24^.	2024	EM	1 EM per thumb, index, dorsal wrist	Double	Yes (1)
			EM	1 EM per thumb, index, dorsal wrist	Double	Yes (6)
			DK	Internal device recordings	Double	Yes*
24	Horeman, T^92^.	2012	Force	1 force sensor under phantom	Double	No
25	Hung, A.J^10^.	2019	DK	Internal device recordings	Double	–
26	Hung, A.J^38^.	2018	DK	Internal device recordings	Double	–
27	Hung, A.J^68^.	2022	DK	Internal device recordings	Double	–
28	Itzkovich, D^25^.	2019	DK	Internal device recordings	Double	No
			DK	Internal device recordings	Double	–
29	Itzkovich, D^26^.	2022	DK	Internal device recordings	Double	Yes^*^
			DK	Internal device recordings	Double	–
			DK	Internal device recordings	Double	Yes (-)
30	Jiang, J^73^.	2017	EM	1 EM per robotic instrument tip	Double	No
31	Jog, A^67^.	2011	DK	Internal device recordings	Double	–
32	Kelly, J.D^40^.	2020	DK	Internal device recordings	Double	–
33	Khan, A^86^.	2020	Accelerometer	1 accelerometer on forceps, 1 accelerometer on needle holder	Double	–
34	King, R.C^16^.	2009	Accelerometer, Flex, Bend	Glove: 2 accelerometers on fingers 2-3, 1 accelerometer on fingers 1, 4 and dorsal hand, 1 bend sensor in palm	Single	No
					Single	No
35	Korte, C^47^.	2021	DK	Internal device recordings	Double	–
36	Laverde, R^88^.	2018	IMU (Apple watch)	1 IMU (Apple watch) per wrist	Double	No
37	Lea, C^65^.	2016	DK	Internal device recordings	Double	No
38	Lee, E.J^19^.	2019	EM, RGB cam.	1 EM per laparoscopic handle, 1 EM on imaging tip of ultrasound transducer	Double	–
					Double	–
39	Li, K^51^.	2020	DK	Internal device recordings	Double	No
40	Lin, H.C^54^.	2006	DK	Internal device recordings	Double	–
41	Lin, Z^89^.	2011	IMU	1 IMU per head, back, upper arms, forearms, hands	Double	No
42	Lin, Z^87^.	2013	IMU	1 IMU per head, back, upper arms, forearms, hands	Double	Yes (2)
43	Liu, J^34^.	2023	DK, RGB cam.	Internal device recordings	Double	–
44	Long, Y^27^.	2021	DK, RGB cam.	Internal device recordings	Double	No
			DK, RGB cam.	Internal device recordings	Double	–
45	Loukas, C^75^.	2013	EM	1 EM per laparoscope handle	Double	No
46	Loukas, C^77^.	2011	EM	1 EM per laparoscopic handle	Double	No
47	Loukas, C^78^.	2013	EM	1 EM per laparoscopic handle	Double	No
48	Lyman, W.B^52^.	2021	DK	Internal device recordings	Double	No
49	Megali, G^48^.	2006	DK	Internal device recording	Double	–
50	Meißner, C^84^.	2014	RFID, Accelerometer	1 RFID tag per instrument (9 total), 1 accelerometer per dorsal hand and wrist	Double	No
51	Murali, A^66^.	2016	DK, RGB cam.	Internal device recordings	Double	No
62	Nguyen, X.A^17^.	2019	IMU	1 IMU per dorsal hand	Double	Yes (1)
			DK	Internal device recordings	Double	No
53	Oquendo, Y.A^71^.	2018	EM, Flex	1 EM per laparoscopic tool, 1 EM on endoscope lens, 1 flex sensor per laparoscopic handle	Double	No
54	Pachtrachai, K^30^.	2021	DK, RGB cam.	Internal device recordings	Double	No
			DK, RGB cam.	Internal device recordings	Double	No
55	Peng, W^62^.	2019	DK	Internal device recordings	Double	No
56	Qin, Y^28^.	2020	DK, RGB cam.	Internal device recordings	Double	No
			DK, RGB cam.	Internal device recordings	Double	–
57	Qin, Y^29^.	2020	DK, RGB cam.	Internal device recordings	Double	No
			DK, RGB cam.	Internal device recordings	Double	–
58	Reiley, C.E^60^.	2010	DK	Internal device recordings	Double	–
59	Rocha, C.D^31^.	2019	DK, RGB cam.	Internal device recordings	Double	–
			DK, RGB cam.	Internal device recordings	Double	–
			DK, RGB cam.	Internal device recordings	Double	–
60	Rosen, J^33^.	2001	Force	1 force sensor on laparoscope handle, 1 force sensor under surgeon’s thumb	Double	No
61	Sabique, P.V^35^.	2023	DK, Force, RGB cam.	Internal device recordings, 1 force sensor on surgical tool holder	Single	–
62	Sang, H^57^.	2016	DK, IMU	Internal device recordings, 1 IMU on robotic control manipulator	Single	–
63	Sberini, L^90^.	2018	IMU, Flex	Glove: 14 flex sensors on finger joints, 1 IMU on dorsal hand	Single	No
64	Sewell, C^69^.	2008	DK	Internal device recordings (simulator)	Double	No
65	Shu, X^56^.	2021	DK	Internal device recordings	Double	–
66	Soangra, R^13^.	2022	EMG, Accelerometer	1 EMG + accelerometer per bicep brachii, tricep brachii, anterior deltoid, flexor carpi ulnaris, extensor carpi ulnaris, thenar eminence	Double	–
67	Song, W^80^.	2006	EM, Force, RGB cam.	1 EM of sheath of scalpel, 1 force sensor on scalpel handle	Single	–
68	Su, H^58^.	2019	DK, Force	Internal device recordings, 1 force sensor at robotic end effector	Single	–
69	Sun, Z^83^.	2018	EM	8 EM arranged around the site	Not applicable	–
70	Tatinati, S^95^.	2015	Accelerometer, IR cam.	IR stylus, 3 accelerometers on tremor compensation instrument	Single	–
71	Tatinati, S^94^.	2017	Accelerometer, IR cam.	IR stylus, 4 accelerometers on tremor compensation instrument	Single	–
72	Topalli, D^49^.	2019	DK	Internal device recordings	Double	No
73	Uemura, M^41^.	2018	EM	1 EM per laparoscopic tool tip	Double	–
74	Wang, Z.H^43^.	2018	DK	Internal device recordings	Double	No
75	Wang, Z^44^.	2018	DK	Internal device recordings	Double	No
76	Wang, Z^82^.	2022	EM	1 EM per instrument tip	Double	–
77	Watson, R.A^91^.	2014	IMU	1 IMU on dorsal right hand	Single	No
78	Xu, J^93^.	2023	Force	1 force sensor on thumb	Single	No
79	Xu, W^79^.	2017	EM	1 EM on manipulator tip	Single	–
80	Zhang, D^20^.	2020	DK, RGB cam.	Internal device recordings	Double	–
			DK	Internal device recordings	Double	No
81	Zhao, H^59^.	2018	DK, RGB cam.	Internal device recordings	Double	No
82	Zheng, Y^74^.	2022	EM	1 EM per laparoscopic handle	Double	Yes (1)
83	Zia, A^37^.	2019	DK, RGB cam.	Internal device recordings	Double	–
84	Zia, A^18^.	2018	Accelerometer, RBG cam.	Knot tying: 1 accelerometer per dorsal wrist	Double	–
				Suturing: 1 accelerometer on dominant wrist, 1 accelerometer on needle holder	Single	–

This table provides an overview of the sensor types and combinations used in the included studies, their placement, and information on the inclusion of both left and right hands, as well as hand dominance.

**Sensor Types and Placement:** *cam*. Camera. **Left-handed:** *(n)* number of surgeons included, *hyphen (-)* no information supplied, *asterisk* ^*^ Not in original dataset, but achieved via data augmentation.

When examining the influence of surgeon handedness, the dataset showed a predominance of right-handedness: among 106 experiments, only 10 included left-handed surgeons, 48 deliberately excluded them, and 48 provided no information. However, two studies augmented their data by hand inversion to simulate left-handed surgeons and pseudo-balance their dataset^24,26^. Loukas et al. evaluated task recognition for both left and right hands using a database consisting of right-handed individuals, revealing superior performance on the right hand due to its higher activity level and consequent abundance of data^75^. Two studies used only right-handed sensor gloves for data collection^16,90^. Furthermore, 89 of the 106 experiments analysed data from both hands, while 16 focused solely on one hand.

### Sensor and data challenges

Several challenges were identified regarding sensor usage. Metallic interference affected data collection for both EM sensors^71,76,80,82–84^ and IMUs using magnetometers^17^. Increasing the distance between EM sensors and the magnetic source led to increased tracking error^83^. Some studies used isolation methods to limit EM sensor contact with metal^71,80^. Nguyen et al. excluded magnetometer data from IMU analysis, favouring accelerometer data over gyroscopic data for skill identification^17^. However, precise accelerometer, gyroscope, and magnetometer data are needed to compute roll, pitch, and yaw angles. Errors in these three propagate over time, causing a phenomenon known as drift^96^. Sang et al. experienced drift with their IMU^57^, and Brown et al. note the inability to estimate the yaw angle using acceleration data alone, suggesting future work with additional magnetometers and gyroscopes^85^.

Uncorrelated noise was observed in EM sensors^82,83^ and IMUs^57^. Sun et al. used an artificial neural network (ANN) to address random measurement errors in EM sensors by directly incorporating the sensors’ intrinsic characteristics^83^. Acquisition errors were also noted with EM sensors^74^, robotic kinematics^52^, and video cameras^18^.

EM^71^, flex^71,90^, and force^58,80^ sensors required calibration. Oquendo et al. calibrated their EM and flex sensor after every five participants to ensure correct positioning and angle recording^71^. Sbernini et al. chose to omit calibration of flex sensor voltage to specific angles to save time, instead using raw voltage measurements^90^. For force sensors, Song et al. used an electrical scale for calibration^80^, while Su et al. used singular value decomposition^58^.

Loukas et al. found interpreting waveform non-optical data alone challenging, preferring to have video recordings of the experiments to assist in data interpretation^75^. Sensor data may lack clarity compared to visual data, such as when identifying tools in use^81^. However, video data is also limited by visibility, lighting, image background, and camera placement^81^. An ML model combining video and EM data for tool tracking yielded poorer results on an animal dataset than on a phantom dataset due to blood obstruction of the video input^19^. Zhao et al. found kinematic data better for clustering in tool trajectory segmentation, as video data has unclear detail and less stability^59^. However, they found video data more necessary when analysing non-expert demonstrations. Murali et al. reported similar findings for surgical task segmentation^66^.

Some studies raised concerns about wearability and usability, reporting issues such as sensor detachment^18^ and wire clutter^16,87^.

### Machine learning methods

**Fig. 3:**
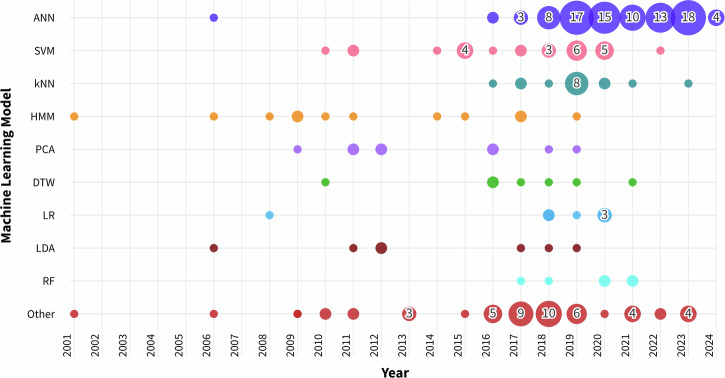
Trends in machine learning model usage in time. Usage trend depiction of various machine learning models, ranging from 2001 to 2024. *HMM* hidden Markov model, *PCA* principal component analysis, *DTW* dynamic time warping, *LR* logarithmic regression, *LDA* linear discriminant analysis, *RF* random forest.

Several studies have explored a variety of ML methods and their combinations. Among these, ANNs were the most popular (91 times), followed by support vector machines (SVM) (26 times), and k-nearest neighbours (kNN) (16 times). While SVMs have received consistent attention since 2010, recent research has increasingly focused on ANNs and other emerging methods (Fig. 3), a trend also observed by Buchlak et al^4^. and Lam et al. ^8^.

The varied goals and outputs of these ML models have led to a wide range of evaluation metrics being used by researchers. Mean accuracy was reported in 69.0% (58/84) of the studies primarily for skill assessment and/or feature detection with only five exceptions^31,59,79,94,95^. Researchers also used metrics such as mean error^14,29,30,47,51,56,83,90^, precision and recall^13,17,21,23,26,31,36,44,61,64,67,74,75,84^, F-1 score^13,17,19,22,24,26,31,34,44,45,61,64,74,81,88,93^, root mean square error^29,35,57,58,79,83^, sensitivity and specificity^36,46,77,91^, area under the curve^26,36,68,70,91,93^, and Jaccard index^18,19,34^. In terms of validation, 82.1% (69/84) of studies detailed their processes, with leave-one-user-out and k-fold splitting being the most common (Table 1).

### ML task: Skill assessment

Surgical skill assessment, which evaluates task execution by surgeons, is the focus of most studies (32/84) (Table 1). Notably, 24 of these were published after 2015.

To train ML methods, surgeon skill levels were established using various assessment measures, such as self-reported experience metrics such as hours^12,20,32,43,51,67^ or years^10,13,38,50^ of experience, number of surgeries performed^39,41,73,87,89,92,93^, or status as a student, resident, or surgeon^46,70,72,90,91^. One study did not specify any criteria for skill^48^. Allen et al. found that some of their included novices were classified as experts by the ML model^70^. Similarly, two other studies found that the “misclassified” novices actually possessed the skills to be considered expert^46,87^.

Eleven studies used objective Global Rating Scale (GRS) systems: the Objective Structured Assessment of Technical Skills (OSATS) system^71,86^; a modified OSATS^12,43,53^; the Global Evaluative Assessment of Robotic Skills (GEARS)^85^; the Global Operative Assessment of Laparoscopic Skills (GOALS)^40^; the Robotic Anastomosis Competence Evaluation tool (RACE)^68^; a Cumulative Sum (CUSUM) analysis-based approach^52^; and custom scoring systems^69,88^. Wang et al. discovered that ML models matched GRS scores more accurately than self-reported skill levels^43^. However, Brown et al. found grading each trial time-consuming and maintaining calibration between reviewers challenging^85^. Kelly et al. only trained their ML model on the top and bottom 15% of graded trials^40^.

Almost half of the experiments (47.2%) are conducted within a robotic surgical context, ten in laparoscopic, and eight in open scenarios. Watson et al. designed a microsurgical vessel anastomosis task^91^. BB models were the most common surgical task (68.6%), particularly prevalent in robotic contexts (41.2%).

As shown in Table 1, motion tracking in 18 experiments used internally logged device kinematic data. Inertial sensors were used in nine experiments, with five using accelerometers^12,13,85–87^ and four using inertial measurement units^88–91^. Magnetic tracking systems were used in five experiments, and EMG sensors in one. Additionally, six studies used mechanical sensors, with four using them alongside other sensor types. Only one study used video footage as additional training data for ML models. However, 14 studies used video recordings to aid human analysis.

Across the 32 studies, 59 algorithm architectures were evaluated. The most common ML algorithm was ANN, appearing 16 times. SVM was used in eight architectures, while LR, RF, and kNN were each used six times. An ensemble approach, combining multiple methods, was noted in 59.4% of cases. Evaluation methods were detailed in 28 studies, with 25 reporting mean accuracy and two reporting mean error. Twelve studies achieved a maximum accuracy rate exceeding 90% (Table 1).

### ML task: Feature detection

Feature detection, which identifies specific surgical tasks or motion components, was the primary focus of 22 studies (Table 1). Except for one, all studies used video, either to contextualise non-optical data or as training input for ML models (Table 2).

RNNs, especially LSTM^14,37,45,74,81^, were the most commonly used ML techniques in this context. Zheng et al. developed a method combining attention-based LSTM to distinguish normal and stressed trials with a simple LSTM to distinguish normal and stressed surgical movements^74^. Zia et al. combined a CNN-LSTM for creating video feature matrices with a separate LSTM for extracting kinematic features^37^. Two studies compared different RNNs for gesture identification^14,81^. Goldbraikh et al. suggested that an ANN for non-optical data could be smaller and faster than one for video data, facilitating easier real-time analysis^81^.

Only 14 studies used ML to break down surgical procedures into actionable steps, with all but two^15,16^ falling into the feature detection category^14,37,45,54,66,75,81,84^. This process, termed surgical process modelling, involves detecting and segmenting surgical steps^97^.

Among the 18 papers reporting mean accuracy^54,74,81,84^, Peng et al. achieved the highest at 97.5%, using a continuous HMM with DTW to segment DK motion data into a labelled sequence of surgical gestures^62^. Precision and recall were also evaluation metrics in six studies^26,61,64,74,75,84^. Loukas et al. achieved the best results, with 89% precision and 94% recall, focusing on surgical phase segmentation^75^.

### ML task: Skill assessment and feature detection

This section of the systematic review covers 13 studies (Table 1). While skill assessment remains the primary focus, interest in utilising feature detection for skill evaluation is growing. Most experiments were conducted in a robotic setting, with BB tasks representing 72.2% of experiment designs. The most commonly used data sources were internal DK data and inertial sensors. Video recordings were utilised in 11 studies, but only one used them as ML input data (Table 2).

Zia et al. used only the OSATS scale to determine surgeon skill level^18^ whereas Nguyen et al. initially categorised participants by the number of procedures performed and then verified eligibility with the OSATS scale^17^. Two studies use the number of hours/surgeries performed^44,49^, four used the year of training or surgeon status^15,36,77,78^, and six did not specify how they determined skill levels^15,16,33,55,60,76^. However, King et al. found novices were more likely to be misclassified as experienced with each task attempt, indicating a learning curve^16^.

Twenty-eight distinct ML architectures were employed, with 60.7% (17/28) involving a feature detection algorithm followed by a skill classifier. Eleven studies used different types of ANNs for feature detection, while 13 employed SVM as the skill classifier. King et al. used HMM for surgical process modelling to classify specific surgical gestures in laparoscopy^16^, and Forestier et al. used SAX-VSM on the JIGSAWS database to classify higher level surgical manoeuvres^15^.

All studies reported mean accuracy except for two^60,78^, and only two provided separate accuracy scores for feature detection and skill assessment^15,44^. The remaining studies focused on identifying the best feature detection ML methods for accurate skill classification. Nguyen et al. achieved the highest overall accuracy of 98.4% when evaluating data from the JIGSAWS database^17^.

### ML task: Tool segmentation and/or tracking

Tool segmentation and/or tracking, which involve accurately identifying and locating surgical instruments within the operative field, are discussed in 11 papers (Table 1). Most studies were conducted in robotic settings, focusing on BB or CM tasks with video input. In laparoscopic settings, Wang et al. conducted BB tasks^82^, while Lee et al. conducted both BB and CM tasks^19^. Three NCS used EM or DK sensors for tool localisation. All studies used ML models involving ANNs, while one also used Gaussian mixture and kNN regression methods^79^.

### ML task: Undesirable motion filtration

Undesirable motion filtration algorithms aim to predict and remove detrimental surgical movement, such as tremors. Three studies focused on this task (Table 1), all conducted through NCS of surgical motion. While all utilised inertial sensors, one also included DK^57^. Two studies gathered training data using infrared technology and validated their tremor estimation and prediction algorithms with real-time accelerometer data^94,95^.

Sang et al. implemented a zero-phase adaptive fuzzy Kalman filter and experimentally validated its effectiveness^57^. Tatinati et al. introduced a moving window-based least squares SVM in 2015^95^, later comparing it to a multidimensional robust extreme learning machine in 2017, achieving up to 81% accuracy^94^.

### ML task: Other studies

The “other” category includes three studies with unique objectives not covered by the previous descriptions (Table 1). Su et al. used an ANN to provide robotic surgeons precise force feedback by measuring the force between tools and tissue, compensating for gravity on the robotic end-effector^58^. Song et al. used a fuzzy NN trained with video, force sensors, and EM tracking inputs to achieve accurate haptic modelling and simulation of surgical tissue cutting^80^. Sabique et al. used RNN methods with DK, force sensors, and video to investigate dimensionality reduction techniques for force estimation in robotic surgery^35^.

### Quality Assessment

The average MERSQI score was 11.0, with scores ranging from 9.5 to 14. The highest achievable score is 18. Many studies were limited in score by their design as single-group studies conducted at a single institution, with outcomes solely from a test setting. The full table of scores can be found in Supplementary Table 1.

## Discussion

This study reviewed the application of ML in analysing surgical motion captured through NOMTS. The findings indicate rapid growth in ML applications for surgical motion analysis and demonstrate the diverse applicability of NOMTS. However, challenges persist in data availability, practical implementation, and model development.

A critical constraint identified is the lack of large, open-source databases. Only 14 experiments used databases with more than 25 participants (Table 1). Most databases remain closed-source, hampering result validation and cross-study comparison. JIGSAWS, a widely-used open-source database, enables comparative analysis. However, its limitation to eight participants restricts the training and testing of ML models, particularly deep learning architectures that require substantial data for effective generalisation^98^.

The predominant reliance on BB task models, due to their ease of execution and data collection, limits the applicability of ML in real surgical contexts. While foundational, BB tasks fail to capture the complexity and unpredictability of real surgical procedures. Nevertheless, there are promising applications in surgical environments: Brown et al. achieved accuracy rates exceeding 90% in porcine prostatectomy experiments^32^, and Ahmidi et al. had similar success in septoplasty procedures^72^. Federated learning could enhance these efforts by enabling the use of decentralised data from multiple institutions while maintaining data privacy^99^. Future research should prioritise developing larger, standardised, open-source databases applicable to real surgical scenarios. This would enable more robust training, benchmarking, and comparison of ML models across diverse surgical environments.

Machine learning methods have shown potential in processing NOMTS data, particularly in detecting subtle patterns in surgical motion that are imperceptible to human observers. The multidimensional, time-series nature of NOMTS data presents challenges for traditional analysis methods. ML approaches like RNNs and transformers are particularly valuable due to their ability to capture sequential dependencies and handle unstructured information^100^.

Selecting appropriate ML models for NOMTS requires careful consideration of data characteristics. RNNs are useful for capturing the sequential nature of surgical motions^101^. CNNs, while traditionally used in image processing, can be adapted to handle spatial aspects of motion data^27,98^. Recent developments in hybrid architectures, such as combining CNNs for local feature extraction with RNNs for global sequence modelling, have shown promise in addressing both spatial and temporal dependencies^37,102^. Transformers offer advantages through parallel data processing, mitigating latency issues common in sequential models, and making them suitable for real-time surgical applications^29^. Additionally, they can capture motion patterns over extended periods^100^. This is important because predictive accuracy in surgery relies on recognising extended sequences of motion rather than just the most recent ones.

Task-specific considerations also influence model selection. Continuous motion prediction benefits from RNNs or hybrid models, while spatial relationship analysis may favour CNNs, such as in tracking the position of instruments. Hybrid models that integrate CNNs and RNNs provide the flexibility to handle both the spatial and temporal dimensions of surgical motion data. For skill assessment, sliding-scale models that move beyond binary classifications of novice or expert would enable more nuanced assessments of surgical ability. Notable insights for trainee education include observations that expert surgeons use certain motion classes less frequently with greater separability between motions^54^, and that needle driving tasks were more relevant for skill differentiation^51^. Furthermore, subjective skill labelling can misrepresent talented beginners and occasional expert errors^43,46,70,87^, leading to inaccurately labelled data and reduced ML model accuracy.

Preprocessing NOMTS data for use with ML models presents challenges. Sensors such as IMUs and EM sensors generate large volumes of high-frequency data with inherent noise^46,54,57,75,84,94,95^. Techniques such as Kalman filtering and down-sampling can help reduce noise and make the data more manageable^87^, but challenges remain for real-time applications.

Surgical procedures generate data from various sources like IMUs, EM sensors, and optical systems, each with different data formats and noise characteristics. Integrating these multimodal data streams into a coherent framework that supports real-time performance is challenging. Recent advancements in ML, especially transformer-based architectures, enable the parallel processing of large volumes of multimodal data without sacrificing accuracy or speed^29,100^. This capability is necessary for maintaining real-time performance in NOMTS applications, as it preserves the temporal relationships across different data streams and ensures data synchronisation.

Despite advances in ML, the field still faces challenges related to interpretability. Future research should rationalise decisions on ML model architecture and hyperparameter tuning to enhance interpretability among peers, promote collective advancement in the field, and ensure reproducibility. Improved interpretability would increase human trust in the algorithms. The field of Explainable Artificial Intelligence (XAI) is developing methods to increase the transparency of supervised ML techniques^103^. In the context of non-optical sensor time-series data, explainability techniques predominantly target sequence classification models. However, there is insufficient research addressing explainability in probabilistic regression models^104^.

ML holds potential for integration into clinical practice. Further development of training algorithms for future surgeons could reduce training time and identify underdeveloped skills. Intelligent surgical systems could also be developed as decision support tools, thereby reducing fatigue and improving outcomes. An underexplored area is the use of ML for surgical process modelling, which could reveal insights and patterns missed by humans, furthering understanding of these processes^97^. Utilising ML to split tasks into smaller granularity levels is a first step. The JIGSAWS database could be a good starting point as it provides labelled manoeuvres and gestures^14,15^.

While ML can enhance surgical performance and reduce the required training time, it should be viewed as an augmentation tool rather than a replacement for clinical expertise. Despite rapid advancements in technology and ML models, their utility is limited by the data they are trained on and may struggle in new, unforeseen situations. Given the complexities of medical practice, broader ML applications face challenges in effective implementation.

Over a third of studies (30/84) show accuracy rates exceeding 90%, demonstrating the potential effectiveness of ML in surgical motion analysis. However, this also highlights the early stage of development in this field.

**Fig. 4:**
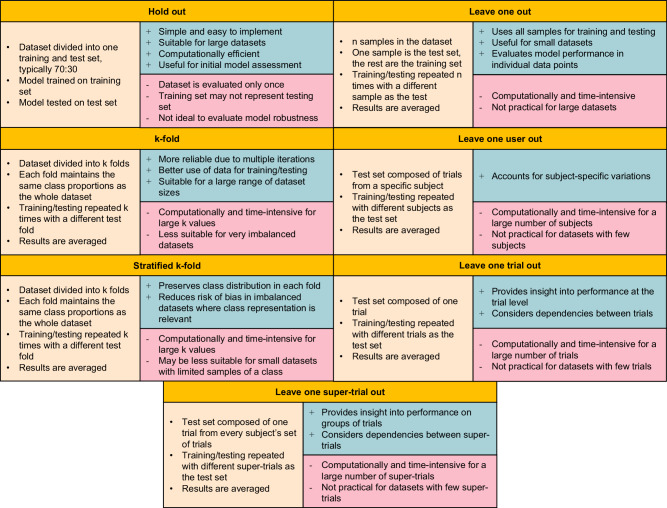
Cross-validation techniques. Cross-validation techniques presented as technique description, *(plus sign +)* advantages, and *(minus sign -)* disadvantages. Consists of hold out^17,19,28,31,33,43,44,46,51,59,73,82,85^, k-fold^10,15,18,27,37,42,49,52,53,57,70,79,84,89,91^, stratified k-fold^29^, leave-one-out^21,35,37,49,62,64,72,87,88,90,92,93,95^, leave one user out^18,20,23,31,34,35,39,41,42,48,56,68,74,75,77,89,96^, leave one trial out^26,75,89^, leave one super-trial out^26,35,36,38,39,46,47,56,58^.

In 79/84 studies, at least one performance metric was reported, and 69/84 provided information on the validation process of ML models. There is notable diversity in assessment and validation techniques due to different applications (Fig. 4). Studies focusing on skill assessment or feature detection typically report accuracy rates, while other categories use a wide range of metrics, posing challenges for cross-model comparisons. Standardising methods is challenging due to variations in database structures and the different approaches required by ML models. A potential solution is standardised benchmark datasets, such as JIGSAWS, enabling researchers to compare and evaluate models effectively.

NOMTS offer benefits in surgical motion analysis. Prioritising research to address implementation challenges and find effective solutions is necessary to unlock their potential in surgical practice.

Synchronisation of multiple data sources is necessary for accurate, reliable, and useful data. It allows precise event sequencing, time series analysis, direct comparison between measurements, and facilitates temporal correlation by linking data from multiple sensors to specific events. This can be done by aligning common events observed in multiple data streams, but it may lead to timestamp misalignment. Fixing desynchronisation post-hoc may render data unusable if metadata is not available to synchronise timestamps across multiple sensor streams. A reliable approach is synchronisation upon acquisition^105^. This may motivate analysing robotic device kinematic data, as the system outputs consistent timestamps.

Manual annotation of events was often required for useful data; however, this was also seen for optical data^18,19,37,80^. Adding an optical data source may help interpret as non-visual data, which is not easily interpreted^75^.

Magnetic interference poses a challenge for IMUs and EM sensors, particularly in environments with metal and electronic equipment like operating rooms. Some studies isolated their tracking systems^71,80^ or avoided using magnetometers to address this issue^17,90^. While reducing magnetic interference in experimental settings may be feasible, addressing inaccuracies in clinical settings remains difficult. Future research should focus on developing solutions to mitigate these inaccuracies.

Variation in sensor placement is observed across studies and even within the same study^18^. Only three studies investigated the optimal sensor placement to maximise accuracy and minimize data volume^13,16,87^. The lack of consistency suggests further research into comparing sensor placement within trials to determine the best positioning. Improper sensor attachment could cause jerking and noise in the data^18^, highlighting the importance of secure attachment methods for consistent and accurate sensor placement to maintain data quality. Excluding left-handed data undermines non-bias and inclusivity, neglecting many left-handed or ambidextrous surgeons. Incorporating this data or using data augmentation techniques prevents biased outcomes and enhances generalisation to real-life scenarios. It also enables the development of more effective surgical tools and techniques, improving patient outcomes.

Integrating NOMTS into surgical practice faces notable legal and practical constraints. Devices used in operating rooms must undergo rigorous medical certification and not disrupt the surgical process. Incorporating NOMTS directly into surgical instruments, as seen in certain robotic and laparoscopic devices^10,37,38^, may offer a solution. One study used a force-sensing forceps with regulatory approval^36^, and EM systems are already used in catheter procedures^106^ and experimentally in live surgery^72^, suggesting that the adoption of NOMTS in surgery may be closer than anticipated.

Due to taxonomy variability within the ML field, not all relevant publications may have been identified. To mitigate this, the authors created search terms with an information specialist, utilised multiple databases spanning medical and technical domains, and explored references from included studies. As only English publications were included, potential language bias may exist.

The possibility of publication bias should be noted, as significant and positive work is more likely to be published^107,108^. Research with poor results often goes unpublished, possibly leading to an absence of failed attempts in this review. Grey literature was excluded to maintain data quality^109^, potentially omitting some valuable works. The scientific community should publish failed attempts and conference presentations, as these contribute to understanding in the field.

In conclusion, the integration of NOMTS and ML in surgical motion analysis represents a promising frontier for surgical advancement. The challenges outlined by this review serve as a roadmap for future research and highlight the importance of collaborative interdisciplinary efforts to shape the future of surgical training and performance.

## Methods

### Search strategy

A comprehensive literature search was conducted across several databases: Embase.com, MEDLINE ALL via Ovid, Web of Science Core Collection, CINAHL via EBSCOhost, and Scopus. The search strategy was developed and implemented by an experienced medical information specialist (WMB) at Erasmus Medical Center on August 23 2024. It was based on three primary concepts: (1) machine learning and artificial intelligence; (2) motion tracking; (3) surgery and surgeon. The search query, detailed in Supplementary Note 1, included relevant terms and their synonyms. All retrieved records were imported into EndNote software, where duplicates were removed using an established method^110^. Additionally, relevant supplementary references identified through backward snowballing bibliographic cross-referencing during the full-text screening stage were considered for further analysis^111^. The review and research protocol were not registered prior to study commencement.

### Study selection

The inclusion criteria required the use of ML techniques to analyse surgical motion data acquired through NOMTS, either independently or in conjunction with optical tracking. In this work, surgical motion is defined as deliberate hand and/or instrument movements performed by surgeons to accomplish surgical tasks. This includes basic tasks like suturing and knot-tying, simulations, and real-life surgeries. Original studies published in peer-reviewed journals, written in English, and available in full-text were assessed for eligibility. Additionally, conference papers from three high-profile medical engineering conferences were included: the International Conference on Intelligent Robots and Systems, the International Conference on Robotics and Automation, and the Conference of the IEEE Engineering in Medicine and Biology Society. Reviews, case-reports, and commentaries were excluded, as well as publications prior to the year 2000 due to their dated relevance. The first reviewer (TZC) screened titles and abstracts to determine eligibility, and full-text versions of selected studies were sought for in-depth review. Any papers lacking an immediate determination of eligibility underwent a secondary review by other reviewers (CT, MG, DV).

### Data extraction process

The primary objective of the systematic review was to outline the types and applications of ML models using NOMTS for surgical motion analysis and to pinpoint future directions for the field, addressing any challenges identified. Secondary objectives included identifying the surgical approach, setting, procedure type, and dataset composition. Additionally, the study aimed to identify the roles of optical sensors when used alongside NOMTS, evaluate the effectiveness of ML models in achieving their tasks, and document the performance metrics and cross-validation techniques employed. All study characteristics and outcome measures were extracted by the first reviewer (TZC).

### Quality assessment

The Medical Education Research Study Quality Instrument (MERSQI)^112^ was used for quality and risk of bias assessment. The tool consists of six domains of study quality: (1) study design, (2) sampling, (3) type of data, (4) validity of evaluation instrument, (5) data analysis, (6) outcomes. Each domain has a maximum score of 3, leading to an overall maximum score of 18. The included articles were scored by the first reviewer (TZC).

## Supplementary information

Supplementary Information

## Data Availability

The data extracted during the current study is available from the corresponding author upon reasonable request.

## Code availability

No code was used for this study.
