# Supplementary material for: Systematic review of machine learning applications using nonoptical motion tracking in surgery
Source: NPJ Digit Med. 2025 Jan 14;8:28. doi: 10.1038/s41746-024-01412-1 (PMC11733004; doi:10.1038/s41746-024-01412-1)
Supplement: Supplementary file 1 — Supplementary Information [file 41746_2024_1412_MOESM1_ESM.pdf]

Supplementary Note 1: Search string and individual database results

Database searched	Platform	Years of coverage	Records	Records after duplicates removed
Medline ALL	Ovid	1946 - Present	1403	1399
Embase	Embase.com	1971 - Present	1798	1064
Web of Science Core Collection*	Web of Knowledge	1975 - Present	1255	638
CINAHL	EBSCO	1982 - Present	92	8
Scopus	Scopus.com	1823 - Present	1406	491
**Total**			**5952**	**3600**

*Science Citation Index Expanded (1975-present); Social Sciences Citation Index (1975-present); Arts & Humanities Citation Index (1975-present); Conference Proceedings Citation Index- Science (1990-present); Conference Proceedings Citation Index- Social Science & Humanities (1990-present); Emerging Sources Citation Index (2005-present)

No other database limits were used than those specified in the search strategies.

Medline ALL Ovid

(exp Artificial Intelligence/ OR exp Machine Learning/ OR Expert Systems/ OR Bayes Theorem/ OR Markov Chains/ OR ((artificial* ADJ3 intelligen*) OR ((artificial* OR back-propagat* OR convolution* OR cellular* OR Cohen-Grossberg* OR complex-valued* OR deep* OR discrete-time* OR feed-forward* OR fractional-order* OR functional-link* OR memristive* OR pulse-coupled* OR quaternion-valued* OR radial-basis-funct* OR reaction-diffus* OR recurrent* OR residual* OR spiking* OR Long-Short-Term-Memory) ADJ3 (neural-network*)) OR ((feature*) ADJ3 (detection OR extraction OR learning OR ranking OR selection)) OR (Ambient ADJ3 intelligen*) OR ((relevance OR support) ADJ3 vector-machine*) OR (automated ADJ3 (recognition OR reasoning)) OR ((classification* OR detection* OR learning) ADJ3 algorithm*) OR Bayesian-model* OR ((Bayesian OR network OR machine OR deep OR supervised OR unsupervised OR Imitation OR Time-Ser*) ADJ3 learning*) OR (Time-Ser* ADJ3 (forecast* OR predict*)) OR expert-system* OR Knowledge-engineer* OR Sequent*-data* OR Autoencoder* OR Auto-encoder* OR back-propagat* OR classifier* OR computer-heuristic* OR cross-validat* OR data-mining* OR edge-detect* OR fuzzy-system* OR Markov* OR hyperheuristic* OR iterative-closest-point* OR k-nearest-neighbor* OR kernel-method* OR knowledge-discovery* OR least-absolute-shrinkage-and-selection-operator* OR memristor* OR metaheuristic* OR molecular-docking* OR multicriteria-decision-analy* OR multifactor-dimensionality-reduct* OR online-analytical-processing* OR outlier-detect* OR perceptron* OR radial-basis-funct* OR random-forest* OR recursive-feature-eliminat* OR recursive-partitioning* OR rough-set* OR (automat* ADJ3 (measure*))).ab,ti,kw. OR ((automat* ADJ3 (measure* OR assess* OR capture*)) OR (objective* ADJ3 evaluat*) OR algorithm* OR ((Gaussian* OR multivariate* OR multi-variate* OR autoregress*) ADJ3 model*)).ti.) AND (Accelerometry/ OR (Motion/ AND (Wearable Electronic Devices/)) OR (((motion* OR interaction*) ADJ3 tool*) OR ((motion* OR kinesiolog* OR kinetic* OR kinematic* OR rotation* OR accelerat* OR position* OR Momentum* OR velocit* OR turn OR mechanic* OR orientat* OR haptic* OR angle*) ADJ3 (monitor* OR tracking OR analy* OR recorder* OR capture* OR sensor* OR classificat* OR field OR patch OR segment*)) OR acceleromet* OR gyroscop* OR (inertia* ADJ3 (sensor OR measure*)) OR ((Flex* OR rotation* OR Stretch* OR Tension* OR Bend* OR Pressure* OR Exoskeleton* OR magnet* OR electromagnet*) ADJ6 (capture* OR measure* OR sensor* OR tracking*)) OR Mocap OR 3D-position* OR Euler-angle* OR quaternion* OR imu OR kalman-filter* OR data-assimilat*).ab,ti,kw. OR (((motion* OR kinesiolog* OR kinetic* OR kinematic* OR rotation* OR accelerat* OR position* OR Momentum* OR velocit* OR turn OR mechanic* OR orientat* OR haptic* OR angle*) AND (monitor* OR tracking OR analy* OR recorder* OR capture* OR sensor* OR classificat* OR field OR patch OR segment*)) OR (wireless ADJ3 sensor*) OR ((performan* OR task-based) ADJ3 metric*)).ti.) AND (General Surgery/ OR exp Surgical Procedures, Operative/ OR exp Surgeons/ OR Surgical Equipment/ OR (surger* OR surgeon* OR surgical* OR operative*).ab,ti,kf.) NOT ((congres* OR abstract*).pt. AND 1800:2019.(sa_year).) AND english.la.

Embase.com

('artificial intelligence'/exp OR 'machine learning'/exp OR 'expert system'/de OR 'Bayes theorem'/de OR 'Markov chain'/de OR ((artificial* NEAR/3 intelligen*) OR ((artificial* OR back-propagat* OR convolution* OR cellular* OR Cohen-Grossberg* OR complex-valued* OR deep* OR discrete-time* OR feed-forward* OR fractional-order* OR functional-link* OR memristive* OR pulse-coupled* OR quaternion-valued* OR radial-basis-funct* OR reaction-diffus* OR recurrent* OR residual* OR spiking* OR Long-Short-Term-Memory) NEAR/3 (neural-network*)) OR ((feature*) NEAR/3 (detection OR extraction OR learning OR ranking OR selection)) OR (Ambient NEAR/3 intelligen*) OR ((relevance OR support) NEAR/3 vector-machine*) OR (automated NEAR/3 (recognition OR reasoning)) OR ((classification* OR detection* OR learning) NEAR/3 algorithm*) OR Bayesian-model* OR ((Bayesian OR network OR machine OR deep OR supervised OR unsupervised OR Imitation OR Time-Ser*) NEAR/3 learning*) OR (Time-Ser* NEAR/3 (forecast* OR predict*)) OR expert-system* OR Knowledge-engineer* OR Sequent*-data* OR Autoencoder* OR Auto-encoder* OR back-propagat* OR classifier* OR computer-heuristic* OR cross-validat* OR data-mining* OR edge-detect* OR fuzzy-system* OR Markov* OR hyperheuristic* OR iterative-closest-point* OR k-nearest-neighbor* OR kernel-method* OR knowledge-discovery* OR least-absolute-shrinkage-and-selection-operator* OR memristor* OR metaheuristic* OR molecular-docking* OR multicriteria-decision-analy* OR multifactor-dimensionality-reduct* OR online-analytical-processing* OR outlier-detect* OR perceptron* OR radial-basis-funct* OR random-forest* OR recursive-feature-eliminat* OR recursive-partitioning* OR rough-set* OR (automat* NEAR/3 (measure*))):ab,ti,kw OR ((automat* NEAR/3 (measure* OR assess* OR capture*)) OR (objective* NEAR/3 evaluat*) OR algorithm* OR ((Gaussian* OR multivariate* OR multi-variate* OR autoregress*) NEAR/3 model*)):ti) AND ('motion analysis system'/de OR accelerometer/de OR accelerometry/de OR gyroscope/de OR 'inertial sensor'/de OR (Motion/de AND ('wearable sensor'/de)) OR 'kalman filter'/de OR 'kalman filtering'/de OR 'kalman filter algorithm'/de OR 'data assimilation'/de OR (((motion* OR interaction*) NEAR/3 tool*) OR ((motion* OR kinesiolog* OR kinetic* OR kinematic* OR rotation* OR accelerat* OR position* OR Momentum* OR velocit* OR turn OR mechanic* OR orientat* OR haptic* OR angle*) NEAR/3 (monitor* OR tracking OR analy* OR recorder* OR capture* OR sensor* OR classificat* OR field OR patch OR segment*)) OR acceleromet* OR gyroscop* OR (inertia* NEAR/3 (sensor OR measure*)) OR ((Flex* OR rotation* OR Stretch* OR Tension* OR Bend* OR Pressure* OR Exoskeleton* OR magnet* OR electromagnet*) NEAR/6 (capture* OR measure* OR sensor* OR tracking*)) OR Mocap OR 3D-position* OR Euler-angle* OR quaternion* OR imu OR kalman-filter* OR data-assimilat*):ab,ti,kw OR (((motion* OR kinesiolog* OR kinetic* OR kinematic* OR rotation* OR accelerat* OR position* OR Momentum* OR velocit* OR turn OR mechanic* OR orientat* OR haptic* OR angle*) AND (monitor* OR tracking OR analy* OR recorder* OR capture* OR sensor* OR classificat* OR field OR patch OR segment*)) OR (wireless NEAR/3 sensor*) OR ((performan* OR task-based) NEAR/3 metric*)):ti) AND (surgery/exp OR surgeon/exp OR 'surgical training'/exp OR 'surgical equipment'/exp OR (surger* OR surgeon* OR surgical* OR operative*):Ab,ti,kw) NOT ([conference abstract]/lim AND [2000-2019]/py) AND [english]/lim

Web of Science

(TS=((artificial* NEAR/3 intelligen*) OR ((artificial* OR back-propagat* OR convolution* OR cellular* OR Cohen-Grossberg* OR complex-valued* OR deep* OR discrete-time* OR feed-forward* OR fractional-order* OR functional-link* OR memristive* OR pulse-coupled* OR quaternion-valued* OR radial-basis-funct* OR reaction-diffus* OR recurrent* OR residual* OR spiking* OR Long-Short-Term-Memory) NEAR/3 (neural-network*)) OR ((feature*) NEAR/3 (detection OR extraction OR learning OR ranking OR selection)) OR (Ambient NEAR/3 intelligen*) OR ((relevance OR support) NEAR/3 vector-machine*) OR (automated NEAR/3 (recognition OR reasoning)) OR ((classification* OR detection* OR learning) NEAR/3 algorithm*) OR Bayesian-model* OR ((Bayesian OR network OR machine OR deep OR supervised OR unsupervised OR Imitation OR Time-Ser*) NEAR/3 learning*) OR (Time-Ser* NEAR/3 (forecast* OR predict*)) OR expert-system* OR Knowledge-engineer* OR Sequent*-data* OR Autoencoder* OR Auto-encoder* OR back-propagat* OR classifier* OR computer-heuristic* OR cross-validat* OR data-mining* OR edge-detect* OR fuzzy-system* OR Markov* OR hyperheuristic* OR iterative-closest-point* OR k-nearest-neighbor* OR kernel-method* OR knowledge-discovery* OR least-absolute-shrinkage-and-selection-operator* OR memristor* OR metaheuristic* OR molecular-docking* OR multicriteria-decision-analy* OR multifactor-dimensionality-reduct* OR online-analytical-processing* OR outlier-detect* OR perceptron* OR radial-basis-funct* OR random-forest* OR recursive-feature-eliminat* OR recursive-partitioning* OR rough-set* OR (automat* NEAR/3 (measure*))) OR TI=(((automat* NEAR/3 (measure* OR assess* OR capture*)) OR

(objective* NEAR/3 evaluat*) OR algorithm* OR ((Gaussian* OR multivariate* OR multi-variate* OR autoregress*) NEAR/3 model*)))) AND (TS=((((motion* OR interaction*) NEAR/3 tool*) OR ((motion* OR kinesiolog* OR kinetic* OR kinematic* OR rotation* OR accelerat* OR position* OR Momentum* OR velocit* OR turn OR mechanic* OR orientat* OR haptic* OR angle*) NEAR/3 (monitor* OR tracking OR analy* OR recorder* OR capture* OR sensor* OR classificat* OR field OR patch OR segment*)) OR acceleromet* OR gyroscop* OR (inertia* NEAR/3 (sensor OR measure*)) OR ((Flex* OR rotation* OR Stretch* OR Tension* OR Bend* OR Pressure* OR Exoskeleton* OR magnet* OR electromagnet*) NEAR/6 (capture* OR measure* OR sensor* OR tracking*)) OR Mocap OR 3D-position* OR Euler-angle* OR quaternion* OR imu OR kalman-filter* OR data-assimilat*)) OR TI=((((motion* OR kinesiolog* OR kinetic* OR kinematic* OR rotation* OR accelerat* OR position* OR Momentum* OR velocit* OR turn OR mechanic* OR orientat* OR haptic* OR angle*) AND (monitor* OR tracking OR analy* OR recorder* OR capture* OR sensor* OR classificat* OR field OR patch OR segment*)) OR (wireless NEAR/3 sensor*) OR ((performan* OR task-based) NEAR/3 metric*)))) AND TS=((surger* OR surgeon* OR surgical* OR operative*)) NOT (DT=(Meeting Abstract OR Meeting Summary) AND py=(1800-2019)) AND LA=(english)

CINAHL EBSCOhost

(MH Artificial Intelligence+ OR MH Machine Learning+ OR MH Expert Systems OR TI((artificial* N2 intelligen*) OR ((artificial* OR back-propagat* OR convolution* OR cellular* OR Cohen-Grossberg* OR complex-valued* OR deep* OR discrete-time* OR feed-forward* OR fractional-order* OR functional-link* OR memristive* OR pulse-coupled* OR quaternion-valued* OR radial-basis-funct* OR reaction-diffus* OR recurrent* OR residual* OR spiking* OR Long-Short-Term-Memory) N2 (neural-network*)) OR ((feature*) N2 (detection OR extraction OR learning OR ranking OR selection)) OR (Ambient N2 intelligen*) OR ((relevance OR support) N2 vector-machine*) OR (automated N2 (recognition OR reasoning)) OR ((classification* OR detection* OR learning) N2 algorithm*) OR Bayesian-model* OR ((Bayesian OR network OR machine OR deep OR supervised OR unsupervised OR Imitation OR Time-Ser*) N2 learning*) OR (Time-Ser* N2 (forecast* OR predict*)) OR expert-system* OR Knowledge-engineer* OR Sequent*-data* OR Autoencoder* OR Auto-encoder* OR back-propagat* OR classifier* OR computer-heuristic* OR cross-validat* OR data-mining* OR edge-detect* OR fuzzy-system* OR Markov* OR hyperheuristic* OR iterative-closest-point* OR k-nearest-neighbor* OR kernel-method* OR knowledge-discovery* OR least-absolute-shrinkage-and-selection-operator* OR memristor* OR metaheuristic* OR molecular-docking* OR multicriteria-decision-analy* OR multifactor-dimensionality-reduct* OR online-analytical-processing* OR outlier-detect* OR perceptron* OR radial-basis-funct* OR random-forest* OR recursive-feature-eliminat* OR recursive-partitioning* OR rough-set* OR (automat* N2 measure*)) OR AB((artificial* N2 intelligen*) OR ((artificial* OR back-propagat* OR convolution* OR cellular* OR Cohen-Grossberg* OR complex-valued* OR deep* OR discrete-time* OR feed-forward* OR fractional-order* OR functional-link* OR memristive* OR pulse-coupled* OR quaternion-valued* OR radial-basis-funct* OR reaction-diffus* OR recurrent* OR residual* OR spiking* OR Long-Short-Term-Memory) N2 (neural-network*)) OR ((feature*) N2 (detection OR extraction OR learning OR ranking OR selection)) OR (Ambient N2 intelligen*) OR ((relevance OR support) N2 vector-machine*) OR (automated N2 (recognition OR reasoning)) OR ((classification* OR detection* OR learning) N2 algorithm*) OR Bayesian-model* OR ((Bayesian OR network OR machine OR deep OR supervised OR unsupervised OR Imitation OR Time-Ser*) N2 learning*) OR (Time-Ser* N2 (forecast* OR predict*)) OR expert-system* OR Knowledge-engineer* OR Sequent*-data* OR Autoencoder* OR Auto-encoder* OR back-propagat* OR classifier* OR computer-heuristic* OR cross-validat* OR data-mining* OR edge-detect* OR fuzzy-system* OR Markov* OR hyperheuristic* OR iterative-closest-point* OR k-nearest-neighbor* OR kernel-method* OR knowledge-discovery* OR least-absolute-shrinkage-and-selection-operator* OR memristor* OR metaheuristic* OR molecular-docking* OR multicriteria-decision-analy* OR multifactor-dimensionality-reduct* OR online-analytical-processing* OR outlier-detect* OR perceptron* OR radial-basis-funct* OR random-forest* OR recursive-feature-eliminat* OR recursive-partitioning* OR rough-set* OR (automat* N2 measure*))) AND (MH Accelerometry OR (MH Motion AND (MH Wearable Sensors)) OR TI(((motion* OR kinesiolog* OR kinetic* OR kinematic* OR rotation* OR accelerat* OR position* OR Momentum* OR velocit* OR turn OR mechanic* OR orientat* OR haptic* OR angle*) N2 (monitor* OR tracking OR analy* OR recorder* OR capture* OR sensor* OR classificat* OR field OR patch)) OR acceleromet* OR gyroscop* OR (inertia* N2 (sensor OR measure*)) OR ((Flex* OR rotation* OR Stretch* OR Tension* OR Bend* OR Pressure* OR Exoskeleton* OR magnet* OR electromagnet*) N5 (capture* OR measure* OR sensor* OR tracking*)) OR Mocap OR 3D-position* OR Euler-angle* OR quaternion* OR imu OR kalman-filter* OR data-assimilat*) OR

AB(((motion* OR kinesiolog* OR kinetic* OR kinematic* OR rotation* OR accelerat* OR position* OR Momentum* OR velocit* OR turn OR mechanic* OR orientat* OR haptic* OR angle*) N2 (monitor* OR tracking OR analy* OR recorder* OR capture* OR sensor* OR classificat* OR field OR patch)) OR acceleromet* OR gyroscop* OR (inertia* N2 (sensor OR measure*)) OR ((Flex* OR rotation* OR Stretch* OR Tension* OR Bend* OR Pressure* OR Exoskeleton* OR magnet* OR electromagnet*) N5 (capture* OR measure* OR sensor* OR tracking*)) OR Mocap OR 3D-position* OR Euler-angle* OR quaternion* OR imu OR kalman-filter* OR data-assimilat*)) AND (MH Surgery, Operative + OR MH Surgeons+ OR MH "Surgical Equipment and Supplies" OR TI(surger* OR surgeon* OR surgical* OR operative*) OR AB(surger* OR surgeon* OR surgical* OR operative*)) AND LA(English) NOT ((MH News OR MH Abstracts OR MH Books+) AND PY 1800-2019)

Scopus

TITLE-ABS-KEY((((artificial* W/2 intelligen*) OR ((artificial* OR back-propagat* OR convolution* OR cellular* OR Cohen-Grossberg* OR complex-valued* OR deep* OR discrete-time* OR feed-forward* OR fractional-order* OR functional-link* OR memristive* OR pulse-coupled* OR quaternion-valued* OR radial-basis-funct* OR reaction-diffus* OR recurrent* OR residual* OR spiking* OR Long-Short-Term-Memory) W/2 (neural-network*)) OR ((feature*) W/2 (detection OR extraction OR learning OR ranking OR selection)) OR (Ambient W/2 intelligen*) OR ((relevance OR support) W/2 vector-machine*) OR (automated W/2 (recognition OR reasoning)) OR ((classification* OR detection* OR learning) W/2 algorithm*) OR Bayesian-model* OR ((Bayesian OR network OR machine OR deep OR supervised OR unsupervised OR Imitation OR Time-Ser*) W/2 learning*) OR (Time-Ser* W/2 (forecast* OR predict*)) OR expert-system* OR Knowledge-engineer* OR Sequent*-data* OR Autoencoder* OR Auto-encoder* OR back-propagat* OR classifier* OR computer-heuristic* OR cross-validat* OR data-mining* OR edge-detect* OR fuzzy-system* OR Markov* OR hyperheuristic* OR iterative-closest-point* OR k-nearest-neighbor* OR kernel-method* OR knowledge-discovery* OR least-absolute-shrinkage-and-selection-operator* OR memristor* OR metaheuristic* OR molecular-docking* OR multicriteria-decision-analy* OR multifactor-dimensionality-reduct* OR online-analytical-processing* OR outlier-detect* OR perceptron* OR radial-basis-funct* OR random-forest* OR recursive-feature-eliminat* OR recursive-partitioning* OR rough-set* OR (automat* W/2 measure*))) AND ((((motion* OR kinesiolog* OR kinetic* OR kinematic* OR rotation* OR accelerat* OR position* OR Momentum* OR velocit* OR turn OR mechanic* OR orientat* OR haptic* OR angle*) W/2 (monitor* OR tracking OR analy* OR recorder* OR capture* OR sensor* OR classificat* OR field OR patch)) OR acceleromet* OR gyroscop* OR (inertia* W/2 (sensor OR measure*)) OR ((Flex* OR rotation* OR Stretch* OR Tension* OR Bend* OR Pressure* OR Exoskeleton* OR magnet* OR electromagnet*) W/5 (capture* OR measure* OR sensor* OR tracking*)) OR Mocap OR 3D-position* OR Euler-angle* OR quaternion* OR imu OR kalman-filter* OR data-assimilat*)) AND ((surger* OR surgeon* OR surgical* OR operative*))) AND NOT DOCTYPE(ab) AND Language(en)

Supplementary Table 1: Medical Education Research Study Quality Instrument (MERSQI)

Author	Year	Study Design	Sampling	Data Type	Validity of Evaluation Instrument	Data Analysis	Outcome	Total
**Skill Assessment**								
Ahmidi, N.[75]	2015	1	2	3	0	2	1.5	9.5
Albasri, S.[26]	2021	1	2	3	2	2	1.5	11.5
Allen, B.[73]	2010	1	2	3	1	3	1.5	11.5
Baghdadi, A.[53]	2020	1	2	3	0	3	1.5	10.5
Bissonnette, V.[49]	2019	1	3	3	2	2	1.5	12.5
Brown, J.D.[88]	2017	1	2	3	1	2	1.5	10.5
Brown, K.C.[15]	2020	1	2	3	1	3	1.5	11.5
Chen, A.B.[30]	2021	1	2	3	0	3	1.5	10.5
Fard, M.J.[56]	2018	1	2	3	1	3	1.5	11.5
Horeman, T.[95]	2012	1	2	3	1	3	1.5	11.5
Hung, A.J.[29]	2018	1	2	3	2	3	3	14
Hung, A.J.[10]	2019	1	2	3	1	3	3	13
Hung, A.J.[71]	2022	1	2	3	2	3	3	14
Jiang, J.[76]	2017	1	2	3	1	3	1.5	11.5
Jog, A.[70]	2011	1	2	3	1	2	1.5	10.5
Kelly, J.D.[43]	2020	1	3	3	2	3	1.5	13.5
Khan, A.[89]	2020	1	2	3	1	2	1.5	10.5
Laverde, R.[91]	2018	1	2	3	1	3	1.5	11.5
Li, K.[54]	2020	1	2	3	2	2	1.5	11.5
Lin, Z.[92]	2011	1	2	3	1	3	1.5	11.5
Lin, Z.[90]	2012	1	2	3	2	3	1.5	12.5
Lyman, W.B.[55]	2021	1	1.5	3	1	3	1.5	11
Megali, G.[51]	2006	1	2	3	0	3	1.5	10.5
Oquendo, Y.A.[74]	2018	1	2	3	3	3	1.5	13.5
Sbernini, L.[93]	2018	1	2	3	2	3	1.5	12.5
Sewell, C.[72]	2008	1	2	3	3	3	1.5	13.5
Soangra, R.[33]	2022	1	2	3	1	3	1.5	11.5
Uemura, M.[44]	2018	1	2	3	0	2	1.5	9.5
Wang, Z.H.[46]	2018	1	2	3	3	2	1.5	12.5
Watson, R.A.[94]	2014	1	2	3	1	3	1.5	11.5
Xu, J.[96]	2023	1	2	3	1	2	1.5	10.5
Zhang, D.[38]	2020	1	2	3	1	3	1.5	11.5
**Feature Detection**								
Ahmidi, N.[39]	2017	1	2	3	0	2	1.5	9.5
van Amsterdam, B.[66]	2019	1	2	3	0	2	1.5	9.5
van Amsterdam, B.[48]	2020	1	2	3	1	2	1.5	10.5
van Amsterdam, B.[31]	2022	1	2	3	1	2	1.5	10.5
Despinoy, F.[64]	2016	1	2	3	0	2	1.5	9.5
DiPietro, R.[34]	2019	1	2.5	3	2	2	1.5	12
Fard, M.J.[67]	2016	1	2	3	1	2	1.5	10.5
Gao, Y.[40]	2016	1	2	3	0	2	1.5	9.5

Author	Year	Study Design	Sampling	Data Type	Validity of Evaluation Instrument	Data Analysis	Outcome	Total
Goldbraikh, A.[84]	2022	1	2	3	1	2	1.5	10.5
Goldbraikh, A.[18]	2024	1	2	3	1	2	1.5	10.5
Itzkovich, D.[41]	2019	1	2	3	0	2	1.5	9.5
Itzkovich, D.[19]	2022	1	2	3	0	2	1.5	9.5
Lea, C.[68]	2016	1	2	3	1	2	1.5	10.5
Lin, H.C.[57]	2006	1	2	3	1	2	1.5	10.5
Long, Y.[42]	2021	1	2	3	1	2	1.5	10.5
Loukas, C.[78]	2013	1	2	3	3	3	1.5	13.5
Meißner, C.[87]	2014	1	2	3	0	2	1.5	9.5
Murali, A.[69]	2016	1	2	3	1	2	1.5	10.5
Peng, W.[65]	2019	1	2	3	1	2	1.5	10.5
Qin, Y.[20]	2020a	1	2	3	1	2	1.5	10.5
Zheng, Y.[77]	2022	1	2	3	2	3	1.5	12.5
Zia, A.[28]	2019	1	2	3	1	3	3	13
**Skill Assessment and Feature Detection**								
Anh, N.X.[58]	2020	1	2	3	0	2	1.5	9.5
Baghdadi, A.[27]	2023	1	2	3	1	3	3	13
Ershad, M.[79]	2019	1	2	3	1	2	1.5	10.5
Forestier, G.[35]	2018	1	2	3	2	2	1.5	11.5
King, R.C.[25]	2012	1	2	3	1	2	1.5	10.5
Loukas, C.[80]	2011	1	2	3	2	3	1.5	12.5
Loukas, C.[81]	2013	1	2	3	2	3	1.5	12.5
Nguyen, X.A.[36]	2019	1	2	3	0	2	1.5	9.5
Reiley, C.E.[63]	2010	1	2	3	0	2	1.5	9.5
Rosen, J.[16]	2001	1	2	3	0	3	1.5	10.5
Topalli, D.[52]	2019	1	2	3	1	2	1.5	10.5
Wang, Z.[47]	2018	1	2	3	1	3	1.5	11.5
Zia, A.[37]	2018	1	1.5	3	1	2	1.5	10
**Tool Tracking**								
Korte, C.[50]	2021	1	2	3	1	2	1.5	10.5
Lee, E.J.[17]	2019	1	2	3	0	3	1.5	10.5
Liu, J.[21]	2023	1	2	3	1	3	1.5	11.5
Pachtrachai, K.[32]	2021	1	2	3	1	2	1.5	10.5
Qin, Y.[23]	2020b	1	2	3	1	2	1.5	10.5
Rocha, C.D.[22]	2019	1	2	3	0	2	1.5	9.5
Shu, X.[59]	2021	1	2	3	1	2	1.5	10.5
Sun, Z.[86]	2018	1	2	3	0	2	1.5	9.5
Wang, Z.[85]	2022	1	2	3	1	2	1.5	10.5
Xu, W.[82]	2017	1	2	3	0	2	1.5	9.5
Zhao, H.[62]	2018	1	2	3	0	2	1.5	9.5
**Undesirable Motion Filtering**								
Sang, H.[60]	2016	1	2	3	2	2	1.5	11.5
Tatinati, S.[98]	2015	1	2	3	3	2	1.5	12.5
Tatinati, S.[97]	2017	1	2	3	2	2	1.5	11.5

Author	Year	Study Design	Sampling	Data Type	Validity of Evaluation Instrument	Data Analysis	Outcome	Total
**Other**								
Sabique, P.V.[24]	2023	1	2	3	1	2	1.5	10.5
Song, W.[83]	2006	1	2	3	0	2	1.5	9.5
Su, H.[61]	2019	1	2	3	1	2	1.5	10.5

Supplementary Table 2: PRISMA 2020 Checklist

From: Page MJ, McKenzie JE, Bossuyt PM, Boutron I, Hoffmann TC, Mulrow CD, et al. The PRISMA 2020 statement: an updated guideline for reporting systematic reviews. BMJ 2021;372:n71. doi: 10.1136/bmj.n71

Section and Topic	Item #	Checklist item	Location where item is reported
**TITLE**			
Title	1	Identify the report as a systematic review.	Title
**ABSTRACT**			
Abstract	2	See the PRISMA 2020 for Abstracts checklist.	Word limit insufficient
**INTRODUCTION**			
Rationale	3	Describe the rationale for the review in the context of existing knowledge.	Introduction
Objectives	4	Provide an explicit statement of the objective(s) or question(s) the review addresses.	Introduction
**METHODS**			
Eligibility criteria	5	Specify the inclusion and exclusion criteria for the review and how studies were grouped for the syntheses.	Methods - Study selection
Information sources	6	Specify all databases, registers, websites, organisations, reference lists and other sources searched or consulted to identify studies. Specify the date when each source was last searched or consulted.	Methods – Search strategy
Search strategy	7	Present the full search strategies for all databases, registers and websites, including any filters and limits used.	Supplementary Note 1
Selection process	8	Specify the methods used to decide whether a study met the inclusion criteria of the review, including how many reviewers screened each record and each report retrieved, whether they worked independently, and if applicable, details of automation tools used in the process.	Methods - Study selection
Data collection process	9	Specify the methods used to collect data from reports, including how many reviewers collected data from each report, whether they worked independently, any processes for obtaining or confirming data from study investigators, and if applicable, details of automation tools used in the process.	Methods – Data extraction process
Data items	10a	List and define all outcomes for which data were sought. Specify whether all results that were compatible with each outcome domain in each study were sought (e.g. for all measures, time points, analyses), and if not, the methods used to decide which results to collect.	Methods – Data extraction process
	10b	List and define all other variables for which data were sought (e.g. participant and intervention characteristics, funding sources). Describe any assumptions made about any missing or unclear information.	Methods – Data extraction process

Section and Topic	Item #	Checklist item	Location where item is reported
Study risk of bias assessment	11	Specify the methods used to assess risk of bias in the included studies, including details of the tool(s) used, how many reviewers assessed each study and whether they worked independently, and if applicable, details of automation tools used in the process.	Methods – Data extraction process; Quality assessment
Effect measures	12	Specify for each outcome the effect measure(s) (e.g. risk ratio, mean difference) used in the synthesis or presentation of results.	-
Synthesis methods	13a	Describe the processes used to decide which studies were eligible for each synthesis (e.g. tabulating the study intervention characteristics and comparing against the planned groups for each synthesis (item #5)).	-
	13b	Describe any methods required to prepare the data for presentation or synthesis, such as handling of missing summary statistics, or data conversions.	-
	13c	Describe any methods used to tabulate or visually display results of individual studies and syntheses.	-
	13d	Describe any methods used to synthesize results and provide a rationale for the choice(s). If meta-analysis was performed, describe the model(s), method(s) to identify the presence and extent of statistical heterogeneity, and software package(s) used.	-
	13e	Describe any methods used to explore possible causes of heterogeneity among study results (e.g. subgroup analysis, meta-regression).	-
	13f	Describe any sensitivity analyses conducted to assess robustness of the synthesized results.	-
Reporting bias assessment	14	Describe any methods used to assess risk of bias due to missing results in a synthesis (arising from reporting biases).	-
Certainty assessment	15	Describe any methods used to assess certainty (or confidence) in the body of evidence for an outcome.	-
**RESULTS**			
Study selection	16a	Describe the results of the search and selection process, from the number of records identified in the search to the number of studies included in the review, ideally using a flow diagram.	Figure 1
	16b	Cite studies that might appear to meet the inclusion criteria, but which were excluded, and explain why they were excluded.	-
Study characteristics	17	Cite each included study and present its characteristics.	Table 1
Risk of bias in studies	18	Present assessments of risk of bias for each included study.	Supplementary Table 1

Section and Topic	Item #	Checklist item	Location where item is reported
Results of individual studies	19	For all outcomes, present, for each study: (a) summary statistics for each group (where appropriate) and (b) an effect estimate and its precision (e.g. confidence/credible interval), ideally using structured tables or plots.	Table 1
Results of syntheses	20a	For each synthesis, briefly summarise the characteristics and risk of bias among contributing studies.	Table 1, 2, 3; Supplementary Table 1; Results
	20b	Present results of all statistical syntheses conducted. If meta-analysis was done, present for each the summary estimate and its precision (e.g. confidence/credible interval) and measures of statistical heterogeneity. If comparing groups, describe the direction of the effect.	-
	20c	Present results of all investigations of possible causes of heterogeneity among study results.	-
	20d	Present results of all sensitivity analyses conducted to assess the robustness of the synthesized results.	-
Reporting biases	21	Present assessments of risk of bias due to missing results (arising from reporting biases) for each synthesis assessed.	-
Certainty of evidence	22	Present assessments of certainty (or confidence) in the body of evidence for each outcome assessed.	-
**DISCUSSION**			
Discussion	23a	Provide a general interpretation of the results in the context of other evidence.	
	23b	Discuss any limitations of the evidence included in the review.	Discussion
	23c	Discuss any limitations of the review processes used.	Discussion
	23d	Discuss implications of the results for practice, policy, and future research.	Discussion
**OTHER INFORMATION**			
Registration and protocol	24a	Provide registration information for the review, including register name and registration number, or state that the review was not registered.	Methods – Search strategy
	24b	Indicate where the review protocol can be accessed, or state that a protocol was not prepared.	Methods – Search strategy
	24c	Describe and explain any amendments to information provided at registration or in the protocol.	-
Support	25	Describe sources of financial or non-financial support for the review, and the role of the funders or sponsors in the review.	Acknowledgements
Competing interests	26	Declare any competing interests of review authors.	Competing interests

Section and Topic	Item #	Checklist item	Location where item is reported
Availability of data, code and other materials	27	Report which of the following are publicly available and where they can be found: template data collection forms; data extracted from included studies; data used for all analyses; analytic code; any other materials used in the review.	Data availability
